# Supplementary figures and images for: Associations between meteorological factors and pregnancy complications during different pregnancy trimesters: a multicenter retrospective study in eastern China
Source: PeerJ. 2025 Jun 27;13:e19621. doi: 10.7717/peerj.19621 (PMC12208105; doi:10.7717/peerj.19621)

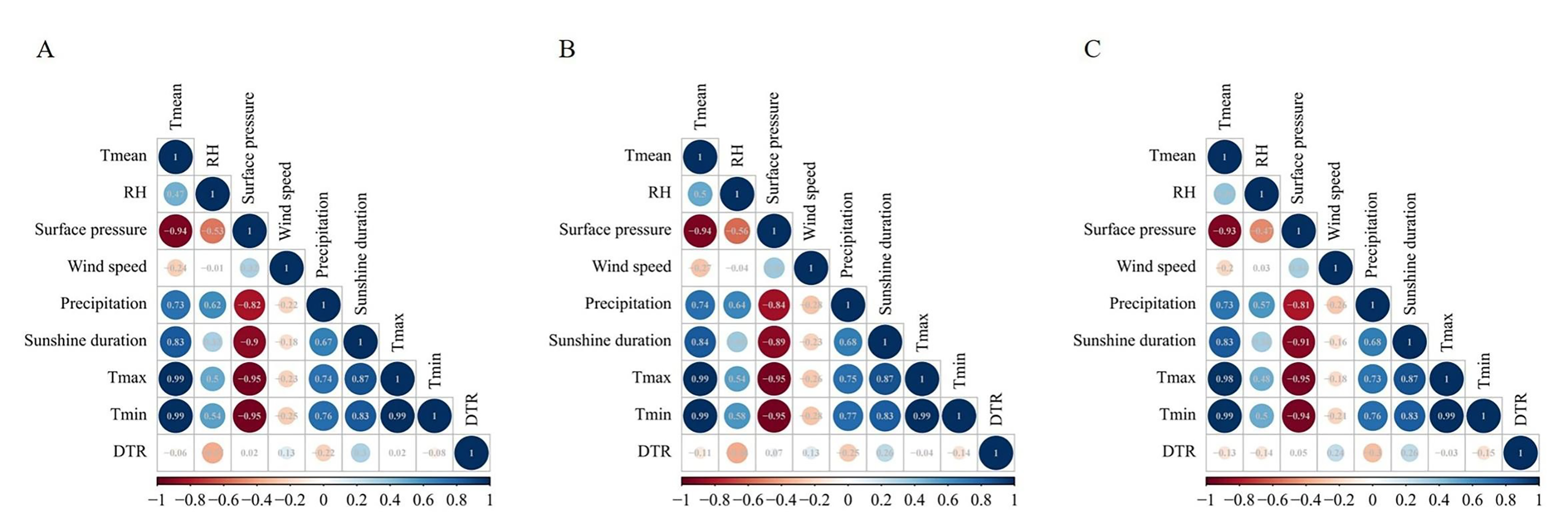

Supplement: Supplemental Information 1 — (A) the first trimester. (B) the second trimester. (C) the first two trimesters. Tmean, daily mean temperature; RH, relative humidity; Tmax, daily maximum temperature; Tmin, daily minimum temperature; DTR, diurnal temperature range. [file peerj-13-19621-s001.png]
