# Supplementary material for: Associations between meteorological factors and pregnancy complications during different pregnancy trimesters: a multicenter retrospective study in eastern China
Source: PeerJ. 2025 Jun 27;13:e19621. doi: 10.7717/peerj.19621 (PMC12208105; doi:10.7717/peerj.19621)
Supplement: Supplemental Information 2 — GDM, gestational diabetes mellitus; SD, standard deviation. [file peerj-13-19621-s002.docx]

**Supplemental Table S1 Maternal characteristics of GDM and non-GDM participants.**

|  | GDM (n = 17814) | Non-GDM (n = 74518) | *P-value* |
| --- | --- | --- | --- |
| Maternal age (years, mean ± SD) | 31.52 ± 4.72 | 29.66 ± 4.46 | < 0.001 |
| Gravidity (n, %) |  |  | < 0.001 |
| 1 | 5312 (29.82) | 26732 (35.87) |  |
| 2 | 4778 (26.82) | 20359 (27.32) |  |
| ≥3 | 7724 (43.36) | 27427 (36.81) |  |
| Parity (n, %) |  |  | < 0.001 |
| Primiparous | 8721 (48.96) | 40527 (54.39) |  |
| Multiparous | 9093 (51.04) | 33991 (45.61) |  |
| Residence (n, %) |  |  | < 0.001 |
| Residents | 8963 (50.31) | 36356 (48.79) |  |
| Immigrants | 8851 (49.69) | 38162 (51.21) |  |
| Fetal gender (n, %) |  |  | 0.675 |
| Male | 9451 (53.06) | 39574 (53.11) |  |
| Female | 8359 (46.92) | 34934 (46.88) |  |
| Missing | 4 (0.02) | 10 (0.01) |  |
| Season of conception (n, %) |  |  | < 0.001 |
| Spring (March–May) | 4740 (26.61) | 17892 (24.01) |  |
| Summer (June–August) | 4310 (24.20) | 16979 (22.78) |  |
| Fall (September–November) | 4055 (22.76) | 19359 (25.98) |  |
| Winter (December–February) | 4709 (26.43) | 20288 (27.23) |  |

GDM, gestational diabetes mellitus; SD, standard deviation.
