# Supplementary material for: Associations between meteorological factors and pregnancy complications during different pregnancy trimesters: a multicenter retrospective study in eastern China
Source: PeerJ. 2025 Jun 27;13:e19621. doi: 10.7717/peerj.19621 (PMC12208105; doi:10.7717/peerj.19621)
Supplement: Supplemental Information 3 — GH, gestational hypertension; SD, standard deviation. [file peerj-13-19621-s003.docx]

**Supplemental Table S2 Maternal characteristics of GH and non-GH participants.**

|  | GH (n = 3860) | Non-GH (n = 88472) | *P-value* |
| --- | --- | --- | --- |
| Maternal age (years, mean ± SD) | 30.80 ± 5.14 | 29.99 ± 4.54 | < 0.001 |
| Gravidity (n, %) |  |  | < 0.001 |
| 1 | 1412 (36.58) | 30632 (34.62) |  |
| 2 | 944 (24.46) | 24193 (27.35) |  |
| ≥3 | 1504 (38.96) | 33647 (38.03) | < 0.001 |
| Parity (n, %) |  |  |  |
| Primiparous | 2174 (56.32) | 47074 (53.21) |  |
| Multiparous | 1686 (43.68) | 41398 (46.79) |  |
| Residence (n, %) |  |  | 0.163 |
| Residents | 1937 (50.18) | 43382 (49.03) |  |
| Immigrants | 1923 (49.82) | 45090 (50.97) |  |
| Fetal gender (n, %) |  |  | 0.800 |
| Male | 2038 (52.80) | 46987 (53.11) |  |
| Female | 1821 (47.18) | 41472 (46.88) |  |
| Missing | 1 (0.02) | 13 (0.01) |  |
| Season of conception (n, %) |  |  | < 0.001 |
| Spring (March–May) | 1121 (29.04) | 21511 (24.31) |  |
| Summer (June–August) | 971 (25.16) | 20318 (22.97) |  |
| Fall (September–November) | 779 (20.18) | 22635 (25.58) |  |
| Winter (December–February) | 989 (25.62) | 24008 (27.14) |  |

GH, gestational hypertension; SD, standard deviation.
