# Supplementary material for: Associations between meteorological factors and pregnancy complications during different pregnancy trimesters: a multicenter retrospective study in eastern China
Source: PeerJ. 2025 Jun 27;13:e19621. doi: 10.7717/peerj.19621 (PMC12208105; doi:10.7717/peerj.19621)
Supplement: Supplemental Information 4 — PE, preeclampsia; SD, standard deviation. [file peerj-13-19621-s004.docx]

**Supplemental Table S3 Maternal characteristics of PE and non-PE participants.**

|  | PE (n = 3101) | Non-PE (n = 89231) | *P-value* |
| --- | --- | --- | --- |
| Maternal age (years, mean ± SD) | 30.90 ± 5.21 | 29.99 ± 4.55 | < 0.001 |
| Gravidity (n, %) |  |  | < 0.001 |
| 1 | 1062 (34.25) | 30982 (34.72) |  |
| 2 | 699 (22.54) | 24438 (27.39) |  |
| ≥3 | 1340 (43.21) | 33811 (37.89) |  |
| Parity (n, %) |  |  | 0.008 |
| Primiparous | 1726 (55.66) | 47522 (53.26) |  |
| Multiparous | 1375 (44.34) | 41709 (46.74) |  |
| Residence (n, %) |  |  | < 0.001 |
| Residents | 1424 (45.92) | 43895 (49.19) |  |
| Immigrants | 1677 (54.08) | 45336 (50.81) |  |
| Fetal gender (n, %) |  |  | 0.001 |
| Male | 1543 (49.76) | 47482 (53.21) |  |
| Female | 1558 (50.24) | 41735 (46.77) |  |
| Missing | 0 | 14 (0.02) |  |
| Season of conception (n, %) |  |  | < 0.001 |
| Spring (March–May) | 884 (28.51) | 21748 (24.37) |  |
| Summer (June–August) | 723 (23.31) | 20566 (23.05) |  |
| Fall (September–November) | 713 (22.99) | 22701 (25.44) |  |
| Winter (December–February) | 781 (25.19) | 24216 (27.14) |  |

PE, preeclampsia; SD, standard deviation.
