# Supplementary material for: Associations between meteorological factors and pregnancy complications during different pregnancy trimesters: a multicenter retrospective study in eastern China
Source: PeerJ. 2025 Jun 27;13:e19621. doi: 10.7717/peerj.19621 (PMC12208105; doi:10.7717/peerj.19621)
Supplement: Supplemental Information 5 — SD, standard deviation. [file peerj-13-19621-s005.docx]

**Supplemental Table S4 Maternal characteristics of hypothyroidism and non- hypothyroidism participants.**

|  | Hypothyroidism (n = 17418) | Non- hypothyroidism (n = 74914) | *P-value* |
| --- | --- | --- | --- |
| Maternal age (years, mean ± SD) | 30.05 ± 4.62 | 30.01 ± 4.56 | 0.068 |
| Gravidity (n, %) |  |  | 0.001 |
| 1 | 6224 (35.73) | 25820 (34.47) |  |
| 2 | 4570 (26.24) | 20567 (27.45) |  |
| ≥3 | 6624 (38.03) | 28527 (38.08) |  |
| Parity (n, %) |  |  | < 0.001 |
| Primiparous | 9845 (56.52) | 39403 (52.60) |  |
| Multiparous | 7573 (43.48) | 35511 (47.40) |  |
| Residence (n, %) |  |  | < 0.001 |
| Residents | 7933 (45.54) | 37386 (49.91) |  |
| Immigrants | 9485 (54.46) | 37528 (50.09) |  |
| Fetal gender (n, %) |  |  | 0.001 |
| Male | 9463 (54.33) | 39562 (52.81) |  |
| Female | 7951 (45.65) | 35342 (47.18) |  |
| Missing | 4 (0.02) | 10 (0.01) |  |
| Season of conception (n, %) |  |  | < 0.001 |
| Spring (March–May) | 4291 (24.64) | 18341 (24.48) |  |
| Summer (June–August) | 4354 (25.00) | 16935 (22.61) |  |
| Fall (September–November) | 4385 (25.17) | 19029 (25.40) |  |
| Winter (December–February) | 4388 (25.19) | 20609 (27.51) |  |

SD, standard deviation.
