# Supplementary material for: Associations between meteorological factors and pregnancy complications during different pregnancy trimesters: a multicenter retrospective study in eastern China
Source: PeerJ. 2025 Jun 27;13:e19621. doi: 10.7717/peerj.19621 (PMC12208105; doi:10.7717/peerj.19621)
Supplement: Supplemental Information 6 — Tmean, daily mean temperature; RH, relative humidity; Tmax, daily maximum temperature; Tmin, daily minimum temperature; DTR, diurnal temperature range; SD, standard deviation; IQR, interquartile range. [file peerj-13-19621-s006.docx]

**Supplemental Table S5 Average exposure levels of meteorological factors during different trimesters in patients with GDM, GH, PE and hypothyroidism and in normal pregnant women.**

| Gestational period | Meteorological factors | GDM | Non-GDM | *P* |  | GH | Non-GH | *P* |  | PE | Non-PE | *P* |  | Hypothyroidism | Non- hypothyroidism | *P* |
| --- | --- | --- | --- | --- | --- | --- | --- | --- | --- | --- | --- | --- | --- | --- | --- | --- |
| The first trimester | T_mean_ (℃) | 17.91 | 17.39 | < 0.001 |  | 18.33 | 17.45 | < 0.001 |  | 17.94 | 17.47 | < 0.001 |  | 17.93 | 17.38 | < 0.001 |
|  | RH (%) | 75.28 | 74.99 | < 0.001 |  | 75.36 | 75.03 | < 0.001 |  | 75.69 | 75.02 | < 0.001 |  | 74.72 | 75.12 | < 0.001 |
|  | Surface pressure (hPa) | 1011.77 | 1012.52 | < 0.001 |  | 1011.29 | 1012.42 | < 0.001 |  | 1011.93 | 1012.39 | < 0.001 |  | 1012.02 | 1012.45 | < 0.001 |
|  | Wind speed (m/s) | 2.88 | 2.93 | < 0.001 |  | 2.91 | 2.92 | 0.450 |  | 2.99 | 2.92 | < 0.001 |  | 2.86 | 2.93 | < 0.001 |
|  | Precipitation (mm) | 5.54 | 5.38 | < 0.001 |  | 5.67 | 5.40 | < 0.001 |  | 5.55 | 5.40 | < 0.001 |  | 5.59 | 5.37 | < 0.001 |
|  | Sunshine duration (hour) | 3.57 | 3.49 | < 0.001 |  | 3.65 | 3.50 | < 0.001 |  | 3.58 | 3.50 | < 0.001 |  | 3.50 | 3.50 | 0.985 |
|  | T_max_ (℃) | 22.60 | 21.96 | < 0.001 |  | 23.08 | 22.04 | < 0.001 |  | 22.67 | 22.06 | < 0.001 |  | 22.42 | 22.01 | < 0.001 |
|  | T_min_ (℃) | 14.89 | 14.26 | < 0.001 |  | 15.33 | 14.34 | < 0.001 |  | 14.93 | 14.37 | < 0.001 |  | 14.83 | 14.28 | < 0.001 |
|  | DTR (℃) | 7.71 | 7.70 | 0.191 |  | 7.75 | 7.70 | < 0.001 |  | 7.74 | 7.70 | 0.007 |  | 7.59 | 7.72 | < 0.001 |
| The second trimester | T_mean_ (℃) | 18.37 | 18.02 | < 0.001 |  | 18.78 | 18.06 | < 0.001 |  | 18.37 | 18.08 | 0.014 |  |  |  |  |
|  | RH (%) | 75.45 | 75.25 | < 0.001 |  | 75.53 | 75.28 | 0.003 |  | 75.74 | 75.27 | < 0.001 |  |  |  |  |
|  | Surface pressure (hPa) | 1011.45 | 1011.76 | < 0.001 |  | 1011.25 | 1011.72 | < 0.001 |  | 1011.72 | 1011.70 | 0.923 |  |  |  |  |
|  | Wind speed (m/s) | 2.85 | 2.89 | < 0.001 |  | 2.88 | 2.88 | 0.713 |  | 2.97 | 2.88 | < 0.001 |  |  |  |  |
|  | Precipitation (mm) | 5.62 | 5.55 | < 0.001 |  | 5.59 | 5.56 | 0.473 |  | 5.57 | 5.56 | 0.869 |  |  |  |  |
|  | Sunshine duration (hour) | 3.56 | 3.56 | 0.653 |  | 3.59 | 3.56 | 0.015 |  | 3.57 | 3.56 | 0.728 |  |  |  |  |
|  | T_max_ (℃) | 23.08 | 22.69 | < 0.001 |  | 23.49 | 22.74 | < 0.001 |  | 23.08 | 22.76 | 0.015 |  |  |  |  |
|  | T_min_ (℃) | 15.41 | 15.00 | < 0.001 |  | 15.81 | 15.04 | < 0.001 |  | 15.40 | 15.06 | 0.012 |  |  |  |  |
|  | DTR (℃) | 7.67 | 7.70 | < 0.001 |  | 7.68 | 7.69 | 0.158 |  | 7.68 | 7.69 | 0.339 |  |  |  |  |
| The first two trimesters | T_mean_ (℃) | 18.15 | 17.72 | < 0.001 |  | 18.57 | 17.77 | < 0.001 |  | 18.17 | 17.79 | < 0.001 |  |  |  |  |
|  | RH (%) | 75.37 | 75.12 | < 0.001 |  | 75.45 | 75.16 | < 0.001 |  | 75.72 | 75.15 | < 0.001 |  |  |  |  |
|  | Surface pressure (hPa) | 1011.60 | 1012.13 | < 0.001 |  | 1011.27 | 1012.06 | < 0.001 |  | 1011.82 | 1012.03 | 0.016 |  |  |  |  |
|  | Wind speed (m/s) | 2.87 | 2.91 | < 0.001 |  | 2.90 | 2.90 | 0.831 |  | 2.98 | 2.90 | < 0.001 |  |  |  |  |
|  | Precipitation (mm) | 5.58 | 5.47 | < 0.001 |  | 5.63 | 5.48 | < 0.001 |  | 5.56 | 5.49 | 0.011 |  |  |  |  |
|  | Sunshine duration (hour) | 3.57 | 3.53 | < 0.001 |  | 3.62 | 3.53 | < 0.001 |  | 3.57 | 3.53 | < 0.001 |  |  |  |  |
|  | T_max_ (℃) | 22.85 | 22.34 | < 0.001 |  | 23.29 | 22.40 | < 0.001 |  | 22.88 | 22.43 | < 0.001 |  |  |  |  |
|  | T_min_ (℃) | 15.16 | 14.64 | < 0.001 |  | 15.58 | 14.71 | < 0.001 |  | 15.17 | 14.73 | < 0.001 |  |  |  |  |
|  | DTR (℃) | 7.69 | 7.70 | 0.009 |  | 7.71 | 7.70 | 0.100 |  | 7.71 | 7.70 | 0.217 |  |  |  |  |

T_mean_, daily mean temperature; RH, relative humidity; T_max_, daily maximum temperature; T_min_, daily minimum temperature; DTR, diurnal temperature range; SD, standard deviation; IQR, interquartile range.
