# Supplementary material for: Associations between meteorological factors and pregnancy complications during different pregnancy trimesters: a multicenter retrospective study in eastern China
Source: PeerJ. 2025 Jun 27;13:e19621. doi: 10.7717/peerj.19621 (PMC12208105; doi:10.7717/peerj.19621)
Supplement: Supplemental Information 7 — Tmean, daily mean temperature; RH, relative humidity; Tmax, daily maximum temperature; Tmin, daily minimum temperature; DTR, diurnal temperature range; SD, standard deviation; IQR, interquartile range. [file peerj-13-19621-s007.docx]

**Supplemental Table S6 Distributions of meteorological factors in different trimesters among participants.**

| Gestational period | Meteorological factors | Mean | SD | Min | 1st | 3rd | 5th | 25th | 50th | 75th | 95th | 97th | 99th | Max | IQR |
| --- | --- | --- | --- | --- | --- | --- | --- | --- | --- | --- | --- | --- | --- | --- | --- |
| The first trimester | T_mean_ (℃) | 17.49 | 6.70 | 6.83 | 7.44 | 7.74 | 8.15 | 11.07 | 17.18 | 23.96 | 27.52 | 28.03 | 28.47 | 28.87 | 12.89 |
|  | RH (%) | 75.04 | 5.01 | 63.48 | 65.32 | 66.49 | 67.31 | 71.42 | 74.39 | 78.46 | 84.22 | 85.26 | 86.56 | 88.72 | 7.04 |
|  | Surface pressure (hPa) | 1012.37 | 7.03 | 1000.04 | 1000.34 | 1000.72 | 1001.20 | 1005.83 | 1013.21 | 1018.95 | 1022.04 | 1022.40 | 1023.13 | 1024.00 | 13.12 |
|  | Wind speed (m/s) | 2.92 | 0.45 | 2.29 | 2.34 | 2.40 | 2.44 | 2.61 | 2.80 | 3.06 | 4.03 | 4.17 | 4.40 | 4.59 | 0.45 |
|  | Precipitation (mm) | 5.41 | 2.31 | 1.10 | 1.66 | 2.13 | 2.36 | 3.67 | 4.97 | 6.74 | 9.82 | 10.34 | 12.26 | 13.13 | 3.08 |
|  | Sunshine duration (hour) | 3.50 | 0.84 | 1.87 | 1.95 | 2.04 | 2.20 | 2.71 | 3.63 | 4.23 | 4.76 | 4.87 | 5.00 | 5.08 | 1.52 |
|  | T_max_ (℃) | 22.08 | 7.39 | 9.80 | 10.40 | 11.01 | 11.42 | 15.03 | 22.07 | 29.18 | 33.01 | 33.62 | 34.60 | 35.19 | 14.14 |
|  | T_min_ (℃) | 14.38 | 7.42 | 1.94 | 2.76 | 3.51 | 3.97 | 7.22 | 14.08 | 21.66 | 25.21 | 25.43 | 25.81 | 26.18 | 14.43 |
|  | DTR (℃) | 7.70 | 0.80 | 5.33 | 5.83 | 6.21 | 6.39 | 7.19 | 7.68 | 8.32 | 8.93 | 9.07 | 9.34 | 10.74 | 1.13 |
| The second trimester | T_mea_n (℃) | 18.09 | 6.53 | 7.17 | 7.76 | 8.14 | 8.48 | 11.69 | 18.49 | 24.35 | 27.27 | 27.76 | 28.19 | 28.45 | 12.66 |
|  | RH (%) | 75.29 | 4.99 | 64.21 | 65.70 | 66.73 | 67.54 | 71.63 | 74.71 | 78.77 | 83.98 | 84.94 | 86.75 | 88.64 | 7.15 |
|  | Surface pressure (hPa) | 1011.70 | 6.94 | 1000.40 | 1000.62 | 1000.97 | 1001.26 | 1005.12 | 1011.89 | 1018.39 | 1021.62 | 1021.99 | 1022.76 | 1023.72 | 13.27 |
|  | Wind speed (m/s) | 2.88 | 0.41 | 2.31 | 2.36 | 2.41 | 2.44 | 2.61 | 2.78 | 2.98 | 3.93 | 4.08 | 4.23 | 4.42 | 0.37 |
|  | Precipitation (mm) | 5.56 | 2.30 | 1.08 | 1.69 | 2.21 | 2.39 | 3.75 | 5.25 | 7.09 | 9.67 | 10.25 | 12.04 | 13.07 | 3.34 |
|  | Sunshine duration (hour) | 3.56 | 0.81 | 1.90 | 1.98 | 2.10 | 2.27 | 2.81 | 3.75 | 4.24 | 4.72 | 4.82 | 4.92 | 5.03 | 1.43 |
|  | T_max_ (℃) | 22.77 | 7.20 | 10.17 | 10.77 | 11.46 | 11.93 | 15.78 | 23.33 | 29.68 | 32.82 | 33.35 | 34.16 | 34.61 | 13.91 |
|  | T_min_ (℃) | 15.07 | 7.26 | 2.32 | 3.07 | 3.85 | 4.44 | 7.95 | 15.51 | 22.15 | 25.14 | 25.32 | 25.60 | 25.85 | 14.20 |
|  | DTR (℃) | 7.69 | 0.76 | 5.34 | 5.94 | 6.26 | 6.43 | 7.20 | 7.64 | 8.27 | 8.89 | 9.03 | 9.27 | 10.55 | 1.06 |
| The first two trimesters | T_mean_ (℃) | 17.80 | 4.58 | 10.22 | 10.95 | 11.26 | 11.48 | 13.18 | 17.82 | 22.36 | 24.15 | 24.50 | 25.05 | 25.22 | 9.19 |
|  | RH (%) | 75.17 | 3.86 | 66.76 | 67.20 | 68.71 | 69.55 | 72.36 | 74.74 | 77.43 | 82.88 | 83.43 | 83.90 | 84.88 | 5.07 |
|  | Surface pressure (hPa) | 1012.02 | 4.87 | 1003.97 | 1004.32 | 1004.73 | 1004.96 | 1007.54 | 1011.92 | 1016.80 | 1019.01 | 1019.52 | 1020.35 | 1020.96 | 9.26 |
|  | Wind speed (m/s) | 2.90 | 0.39 | 2.47 | 2.50 | 2.53 | 2.54 | 2.64 | 2.76 | 3.00 | 3.82 | 4.01 | 4.19 | 4.25 | 0.36 |
|  | Precipitation (mm) | 5.49 | 1.62 | 2.23 | 2.72 | 3.06 | 3.22 | 4.19 | 5.42 | 6.58 | 8.30 | 9.05 | 9.81 | 10.12 | 2.39 |
|  | Sunshine duration (hour) | 3.53 | 0.56 | 2.46 | 2.49 | 2.57 | 2.67 | 3.05 | 3.53 | 4.02 | 4.41 | 4.48 | 4.60 | 4.64 | 0.98 |
|  | T_max_ (℃) | 22.44 | 5.04 | 14.46 | 14.89 | 15.16 | 15.45 | 17.34 | 22.52 | 27.48 | 29.58 | 29.77 | 29.98 | 30.19 | 10.14 |
|  | T_min_ (℃) | 14.74 | 5.08 | 6.09 | 6.56 | 7.33 | 7.69 | 9.65 | 14.82 | 19.84 | 21.83 | 21.94 | 22.29 | 22.54 | 10.18 |
|  | DTR (℃) | 7.70 | 0.52 | 6.43 | 6.63 | 6.82 | 6.92 | 7.31 | 7.66 | 8.02 | 8.67 | 8.78 | 9.16 | 9.48 | 0.71 |

T_mean_, daily mean temperature; RH, relative humidity; T_max_, daily maximum temperature; T_min_, daily minimum temperature; DTR, diurnal temperature range; SD, standard deviation; IQR, interquartile range.
