# Supplementary material for: Associations between meteorological factors and pregnancy complications during different pregnancy trimesters: a multicenter retrospective study in eastern China
Source: PeerJ. 2025 Jun 27;13:e19621. doi: 10.7717/peerj.19621 (PMC12208105; doi:10.7717/peerj.19621)
Supplement: Supplemental Information 8 — Tmean, daily mean temperature; RH, relative humidity; Tmax, daily maximum temperature; Tmin, daily minimum temperature; DTR, diurnal temperature range; SD, standard deviation; IQR, interquartile range. [file peerj-13-19621-s008.docx]

**Supplemental Table S7 Distributions of weekly meteorological factors during the first 24 gestational weeks among participants.**

| Meteorological factors | Mean | SD | Min | 1st | 3rd | 5th | 50th | 95th | 97th | 99th | Max |
| --- | --- | --- | --- | --- | --- | --- | --- | --- | --- | --- | --- |
| T_mean_ (℃) | 17.74 | 7.64 | 1.32 | 3.78 | 4.98 | 6.00 | 17.81 | 29.27 | 30.01 | 31.11 | 33.04 |
| RH (%) | 75.16 | 9.14 | 37.40 | 50.20 | 55.95 | 58.52 | 75.79 | 89.03 | 90.46 | 92.72 | 95.07 |
| Surface pressure (hPa) | 1012.09 | 8.24 | 988.41 | 997.04 | 998.63 | 999.51 | 1012.83 | 1024.61 | 1025.67 | 1027.35 | 1031.99 |
| Wind speed (m/s) | 2.90 | 0.78 | 1.36 | 1.62 | 1.77 | 1.87 | 2.76 | 4.36 | 4.66 | 5.31 | 6.82 |
| Precipitation (mm) | 5.47 | 5.28 | 0.00 | 0.00 | 0.00 | 0.00 | 4.02 | 15.97 | 18.23 | 24.43 | 37.20 |
| Sunshine duration (hour) | 3.53 | 1.16 | 1.27 | 1.45 | 1.62 | 1.75 | 3.47 | 5.52 | 5.75 | 6.09 | 6.60 |
| T_max_ (℃) | 22.37 | 8.59 | 2.71 | 6.00 | 7.57 | 8.57 | 23.00 | 35.71 | 37.00 | 38.43 | 40.43 |
| T_min_ (℃) | 14.68 | 8.44 | -3.71 | -1.29 | 0.57 | 1.43 | 14.57 | 26.71 | 27.29 | 28.00 | 29.14 |
| DTR (℃) | 7.70 | 2.27 | 1.57 | 2.86 | 3.71 | 4.14 | 7.57 | 11.43 | 12.00 | 13.14 | 16.43 |

T_mean_, daily mean temperature; RH, relative humidity; T_max_, daily maximum temperature; T_min_, daily minimum temperature; DTR, diurnal temperature range; SD, standard deviation; IQR, interquartile range.
