# Supplementary material for: Associations between meteorological factors and pregnancy complications during different pregnancy trimesters: a multicenter retrospective study in eastern China
Source: PeerJ. 2025 Jun 27;13:e19621. doi: 10.7717/peerj.19621 (PMC12208105; doi:10.7717/peerj.19621)
Supplement: Supplemental Information 9 [file peerj-13-19621-s009.docx]

**Supplemental Table S8 Distributions of meteorological factors during the first 13 gestational weeks among participants.**

| Meteorological factors | Mean | SD | Min | 1st | 3rd | 5th | 50th | 95th | 97th | 99th | Max |
| --- | --- | --- | --- | --- | --- | --- | --- | --- | --- | --- | --- |
| T_mean_ (℃) | 17.49 | 7.66 | 1.32 | 3.71 | 4.87 | 5.93 | 17.51 | 29.27 | 30.01 | 31.09 | 33.04 |
| RH (%) | 75.05 | 9.13 | 37.40 | 50.20 | 55.95 | 58.49 | 75.72 | 88.92 | 90.44 | 92.72 | 95.07 |
| Surface pressure (hPa) | 1012.37 | 8.22 | 988.41 | 997.19 | 998.78 | 999.68 | 1013.38 | 1024.74 | 1025.81 | 1027.36 | 1031.99 |
| Wind speed (m/s) | 2.92 | 0.79 | 1.36 | 1.63 | 1.78 | 1.89 | 2.77 | 4.41 | 4.69 | 5.33 | 6.82 |
| Precipitation (mm) | 5.41 | 5.22 | 0.00 | 0.00 | 0.00 | 0.00 | 3.98 | 15.82 | 17.93 | 23.81 | 37.20 |
| Sunshine duration (hour) | 3.50 | 1.17 | 1.27 | 1.45 | 1.61 | 1.74 | 3.44 | 5.51 | 5.75 | 6.09 | 6.60 |
| T_max_ (℃) | 22.09 | 8.62 | 2.71 | 6.00 | 7.43 | 8.43 | 22.57 | 35.71 | 37.00 | 38.43 | 40.43 |
| T_min_ (℃) | 14.39 | 8.44 | -3.71 | -1.29 | 0.57 | 1.43 | 14.14 | 26.71 | 27.14 | 27.86 | 29.14 |
| DTR (℃) | 7.70 | 2.28 | 1.57 | 2.86 | 3.71 | 4.14 | 7.57 | 11.57 | 12.00 | 13.14 | 16.43 |

T_mean_, daily mean temperature; RH, relative humidity; T_max_, daily maximum temperature; T_min_, daily minimum temperature; DTR, diurnal temperature range; SD, standard deviation; IQR, interquartile range.
