# Supplementary material for: Associations between meteorological factors and pregnancy complications during different pregnancy trimesters: a multicenter retrospective study in eastern China
Source: PeerJ. 2025 Jun 27;13:e19621. doi: 10.7717/peerj.19621 (PMC12208105; doi:10.7717/peerj.19621)
Supplement: Supplemental Information 10 — OR, odds ratio; 95% CI, 95% confidence interval. All models were adjusted for maternal age, gravidity, parity, season of conception and year of conception. [file peerj-13-19621-s010.docx]

**Supplemental Table S9 Associations between meteorological factor score (per 10 points increase) with pregnancy complications in different trimesters among participants.**

|  | The first trimester | | The second trimester | | The first two trimesters | |
| --- | --- | --- | --- | --- | --- | --- |
|  | OR (95% *CI*) | *p-value* | OR (95% *CI*) | *p-value* | OR (95% *CI*) | *p-value* |
| GDM | 1.232 (1.122, 1.353) | <0.001 | 1.223 (1.069, 1.400) | 0.003 | 1.284 (1.151, 1.433) | <0.001 |
| GH | 1.770 (1.385, 2.262) | <0.001 | 1.469 (1.230, 1.754) | <0.001 | 1.880 (1.515, 2.334) | <0.001 |
| PE | 1.230 (1.051, 1.441) | 0.010 | 0.986 (0.977, 0.996) | 0.008 | 1.123 (1.047, 1.205) | 0.001 |
| Hypothyroidism | 1.604 (1.401, 1.837) | <0.001 |  |  |  |  |

OR, odds ratio; 95% *CI*, 95% confidence interval. All models were adjusted for maternal age, gravidity, parity, season of conception and year of conception.
