# Supplementary material for: Associations between meteorological factors and pregnancy complications during different pregnancy trimesters: a multicenter retrospective study in eastern China
Source: PeerJ. 2025 Jun 27;13:e19621. doi: 10.7717/peerj.19621 (PMC12208105; doi:10.7717/peerj.19621)
Supplement: Supplemental Information 11 — Tmean, daily mean temperature; RH, relative humidity; Tmax, daily maximum temperature; Tmin, daily minimum temperature; DTR, diurnal temperature range. Extremes high meteorological factors were defined by different percentiles (95th, 97th and 99th) of meteorological factors. Distributed lag non-linear models incorporating logistic regression were adjusted for maternal age, gravidity, parity, season of conception and year of conception. [file peerj-13-19621-s011.docx]

**Supplemental Table S10 The windows of susceptibility for effects of extremely high meteorological factors on risks of pregnancy complications.**

| Pregnancy complication | Extremely high meteorological factor | susceptible time window (described by gestational weeks) | | |
| --- | --- | --- | --- | --- |
|  |  | 95th percentile | 97th percentile | 99th percentile |
| GDM | Precipitation (mm) | 14-20 | 13-20 | 14-20 |
|  | Sunshine duration (hour) | 1-8 | 1-7 |  |
| GH | _Tmean_ (℃) | 18-21 | 18-22 | 18-23 |
|  | RH (%) | 15-18 | 15-18 | 16-18 |
|  | Wind speed (m/s) |  |  | 18 |
|  | Precipitation (mm) | 8-19 | 7-19 | 5-15 |
|  | Sunshine duration (hour) | 6-8 |  |  |
|  | T_max_ (℃) | 18-20 | 19-21 | 20-21 |
| PE | RH (%) | 4-9 |  |  |
|  | Surface pressure (hPa) | 9-10 | 10 |  |
|  | Precipitation (mm) | 1-7 and 18-24 | 18-24 |  |
|  | Sunshine duration (hour) |  | 8-18 | 6-20 |
|  | T_max_ (℃) | 23-24 | 23-24 | 10-13 and 23-24 |
| Hypothyroidism | T_mean_ (℃) |  | 3-4 | 2-6 |
|  | RH (%) |  | 1-7 | 1-8 |
|  | Surface pressure (hPa) | 3-4 and 8-9 and 13 | 13 | 2-13 |
|  | Wind speed (m/s) | 2-12 | 2-12 | 5-13 |
|  | Precipitation (mm) | 1-13 | 1-13 |  |
|  | Sunshine duration (hour) |  |  | 10-13 |
|  | T_max_ (℃) | 1-8 | 1-5 | 1-4 |
|  | T_min_ (℃) | 2-4 | 2-4 | 2-4 |
|  | DTR (℃) |  |  | 2-4 |

T_mean_, daily mean temperature; RH, relative humidity; T_max_, daily maximum temperature; T_min_, daily minimum temperature; DTR, diurnal temperature range.

Extremes high meteorological factors were defined by different percentiles (95th, 97th and 99th) of meteorological factors.

Distributed lag non-linear models incorporating logistic regression were adjusted for maternal age, gravidity, parity, season of conception and year of conception.
