# Supplementary material for: Associations between meteorological factors and pregnancy complications during different pregnancy trimesters: a multicenter retrospective study in eastern China
Source: PeerJ. 2025 Jun 27;13:e19621. doi: 10.7717/peerj.19621 (PMC12208105; doi:10.7717/peerj.19621)
Supplement: Supplemental Information 12 — Tmean, daily mean temperature; RH, relative humidity; Tmax, daily maximum temperature; Tmin, daily minimum temperature; DTR, diurnal temperature range. Extremes low meteorological factors were defined by different percentiles (95th, 97th and 99th) of meteorological factors. Distributed lag non-linear models incorporating logistic regression were adjusted for maternal age, gravidity, parity, season of conception and year of conception. [file peerj-13-19621-s012.docx]

**Supplemental Table S11 The windows of susceptibility for effects of extremely low meteorological factors on risks of pregnancy complications.**

| Pregnancy complication | Extremely low meteorological factors | susceptible time window (described by gestational weeks) | | |
| --- | --- | --- | --- | --- |
|  |  | 5th percentile | 3rd percentile | 1st percentile |
| GDM | DTR (℃) | 1 | 1 | 1 |
| GH | Surface pressure (hPa) | 11-14 | 11-13 |  |
|  | Wind speed (m/s) | 10-19 | 10-18 | 10-18 |
|  | T_min_ (℃) |  |  | 9-16 |
| PE | Precipitation (mm) | 18-21 (defined by10th percentile) |  |  |
|  | T_max_ (℃) | 12-14 | 11-14 | 11-14 |
|  | DTR (℃) |  |  | 10-19 |
| Hypothyroidism | T_mean_ (℃) |  | 3-5 and 12-13 | 2-5 and 12-13 |
|  | RH (%) | 1-3 and 13 | 1-3 and 12-13 | 1-4 and 12-13 |
|  | Surface pressure (hPa) | 4-5 | 4-5 | 4 |
|  | Wind speed (m/s) |  |  | 4-7 |
|  | Precipitation (mm) | 1-12 (defined by10th percentile) |  | 3-13 |
|  | T_max_ (℃) | 1 and 12-13 | 1-2 and 12-13 | 1-4 and 11-13 |
|  | T_min_ (℃) | 4-5 | 3-6 and 12-13 | 2-7 |
|  | DTR (℃) | 1-2 | 1-2 and 13 | 1-3 and 11-13 |

T_mean_, daily mean temperature; RH, relative humidity; T_max_, daily maximum temperature; T_min_, daily minimum temperature; DTR, diurnal temperature range.

Extremes low meteorological factors were defined by different percentiles (95th, 97th and 99th) of meteorological factors.

Distributed lag non-linear models incorporating logistic regression were adjusted for maternal age, gravidity, parity, season of conception and year of conception.
