# Supplementary material for: Associations between meteorological factors and pregnancy complications during different pregnancy trimesters: a multicenter retrospective study in eastern China
Source: PeerJ. 2025 Jun 27;13:e19621. doi: 10.7717/peerj.19621 (PMC12208105; doi:10.7717/peerj.19621)
Supplement: Supplemental Information 13 — OR, odds ratio; 95% CI, 95% confidence interval; Tmean, daily mean temperature; RH, relative humidity; Tmax, daily maximum temperature; Tmin, daily minimum temperature; DTR, diurnal temperature range; MFS, meteorological factor score; GDM, gestational diabetes mellitus. Meteorological factor extremes were defined by different percentiles (5th, 3rd, 1st and 95th, 97th, 99th) of meteorological factors. All models were adjusted for maternal age, gravidity, parity, season of conception and year of conception. [file peerj-13-19621-s013.docx]

**Supplemental Table S12 Associations between extreme meteorological factors with GDM in different trimesters among participants.**

| Gestational period | Meteorological factors | Extreme low meteorological factor | | | Extreme high meteorological factor | | |
| --- | --- | --- | --- | --- | --- | --- | --- |
|  |  | 5th | 3rd | 1st | 95th | 97th | 99th |
| The first trimester | T_mean_ (℃) | 0.975 (0.917, 1.036) | 1.001 (0.929, 1.078) | 0.887 (0.814, 0.967) | 0.989 (0.929, 1.053) | 1.058 (0.987, 1.133) | 1.058 (0.973, 1.151) |
|  | RH (%) | 0.928 (0.868, 0.992) | 0.914 (0.853, 0.980) | 0.979 (0.898, 1.066) | 1.066 (0.999, 1.137) | 1.031 (0.962, 1.106) | 1.036 (0.954, 1.126) |
|  | Surface pressure (hPa) | 1.027 (0.968, 1.091) | 0.953 (0.888, 1.023) | 0.962 (0.890, 1.040) | 0.998 (0.936, 1.064) | 0.951 (0.881, 1.028) | 0.926 (0.844, 1.016) |
|  | Wind speed (m/s) | 0.958 (0.903, 1.017) | 0.956 (0.898, 1.018) | 0.988 (0.914, 1.068) | 1.103 (1.000, 1.217) | 1.051 (0.926, 1.192) | 0.896 (0.794, 1.010) |
|  | Precipitation (mm) | 0.990 (0.929, 1.055) | 0.988 (0.922, 1.058) | 1.037 (0.949, 1.132) | 1.028 (0.964, 1.096) | 0.985 (0.921, 1.053) | 1.018 (0.938, 1.103) |
|  | Sunshine duration (hour) | 0.965 (0.900, 1.034) | 0.977 (0.912, 1.046) | 0.948 (0.863, 1.041) | 0.975 (0.914, 1.039) | 1.013 (0.948, 1.082) | 1.133 (1.043, 1.229) |
|  | T_max_ (℃) | 0.944 (0.887, 1.004) | 1.012 (0.939, 1.091) | 0.884 (0.810, 0.964) | 1.016 (0.954, 1.081) | 1.015 (0.947, 1.087) | 1.064 (0.981, 1.155) |
|  | T_min_ (℃) | 0.969 (0.911, 1.031) | 0.993 (0.921, 1.070) | 0.903 (0.827, 0.985) | 1.000 (0.940, 1.064) | 1.008 (0.941, 1.079) | 1.025 (0.946, 1.111) |
|  | DTR (℃) | 0.952 (0.896, 1.011) | 0.947 (0.885, 1.013) | 0.998 (0.914, 1.089) | 0.917 (0.861, 0.976) | 1.001 (0.932, 1.076) | 1.001 (0.919, 1.090) |
| The second trimester | T_mean_ (℃) | 1.022 (0.929, 1.124) | 0.918 (0.825, 1.021) | 1.051 (0.948, 1.164) | 1.102 (1.010, 1.202) | 1.033 (0.932, 1.144) | 0.993 (0.896, 1.101) |
|  | RH (%) | 0.907 (0.827, 0.996) | 0.961 (0.863, 1.070) | 0.955 (0.859, 1.062) | 1.046 (0.958, 1.143) | 1.039 (0.938, 1.150) | 1.060 (0.955, 1.177) |
|  | Surface pressure (hPa) | 1.005 (0.925, 1.092) | 0.944 (0.857, 1.041) | 1.057 (0.959, 1.165) | 0.946 (0.856, 1.046) | 1.004 (0.894, 1.128) | 0.980 (0.877, 1.094) |
|  | Wind speed (m/s) | 0.951 (0.878, 1.031) | 0.983 (0.892, 1.084) | 0.854 (0.770, 0.947) | 0.889 (0.785, 1.006) | 0.839 (0.720, 0.977) | 1.026 (0.909, 1.159) |
|  | Precipitation (mm) | 0.967 (0.884, 1.057) | 0.983 (0.878, 1.100) | 1.041 (0.934, 1.160) | 1.070 (0.982, 1.166) | 0.962 (0.870, 1.064) | 1.129 (1.020, 1.248) |
|  | Sunshine duration (hour) | 0.974 (0.888, 1.068) | 0.963 (0.859, 1.080) | 0.882 (0.789, 0.986) | 0.988 (0.907, 1.076) | 1.048 (0.948, 1.160) | 0.932 (0.840, 1.033) |
|  | T_max_ (℃) | 0.933 (0.845, 1.029) | 0.885 (0.791, 0.989) | 0.946 (0.849, 1.053) | 1.026 (0.945, 1.115) | 1.043 (0.942, 1.155) | 0.945 (0.852, 1.048) |
|  | T_min_ (℃) | 0.987 (0.894, 1.088) | 0.859 (0.766, 0.963) | 0.904 (0.812, 1.007) | 1.037 (0.956, 1.126) | 1.064 (0.964, 1.173) | 0.912 (0.820, 1.013) |
|  | DTR (℃) | 0.914 (0.837, 0.998) | 0.956 (0.855, 1.068) | 0.983 (0.886, 1.091) | 1.052 (0.953, 1.161) | 0.977 (0.871, 1.097) | 0.921 (0.820, 1.036) |
| The first two trimesters | T_mean_ (℃) | 1.059 (0.887, 1.264) | 1.160 (0.981, 1.371) | 0.836 (0.666, 1.048) | 1.036 (0.874, 1.228) | 1.179 (1.000, 1.391) | 1.096 (0.920, 1.305) |
|  | RH (%) | 0.850 (0.701, 1.032) | 1.057 (0.885, 1.262) | 0.919 (0.759, 1.112) | 1.086 (0.913, 1.292) | 1.062 (0.885, 1.274) | 0.960 (0.801, 1.150) |
|  | Surface pressure (hPa) | 0.951 (0.811, 1.116) | 1.175 (1.002, 1.378) | 1.063 (0.906, 1.249) | 1.030 (0.847, 1.252) | 0.948 (0.787, 1.142) | 0.898 (0.732, 1.101) |
|  | Wind speed (m/s) | 1.032 (0.880, 1.210) | 0.957 (0.807, 1.136) | 0.689 (0.574, 0.828) | 0.898 (0.674, 1.197) | 0.907 (0.745, 1.105) | 0.813 (0.652, 1.013) |
|  | Precipitation (mm) | 0.996 (0.826, 1.200) | 1.090 (0.920, 1.291) | 0.886 (0.732, 1.072) | 0.997 (0.849, 1.171) | 1.186 (1.011, 1.392) | 0.942 (0.799, 1.111) |
|  | Sunshine duration (hour) | 0.756 (0.618, 0.925) | 0.912 (0.761, 1.093) | 0.952 (0.787, 1.153) | 1.000 (0.848, 1.180) | 0.809 (0.682, 0.961) | 1.085 (0.909, 1.294) |
|  | T_max_ (℃) | 0.791 (0.651, 0.962) | 1.137 (0.957, 1.350) | 0.881 (0.701, 1.107) | 1.003 (0.852, 1.181) | 0.845 (0.712, 1.004) | 1.124 (0.955, 1.323) |
|  | T_min_ (℃) | 0.810 (0.668, 0.982) | 1.098 (0.911, 1.325) | 0.763 (0.604, 0.963) | 1.031 (0.877, 1.212) | 0.820 (0.691, 0.973) | 1.032 (0.879, 1.212) |
|  | DTR (℃) | 0.826 (0.681, 1.001) | 0.899 (0.745, 1.084) | 0.919 (0.765, 1.104) | 0.959 (0.786, 1.169) | 0.826 (0.679, 1.006) | 0.926 (0.759, 1.131) |

OR, odds ratio; 95% *CI*, 95% confidence interval; T_mean_, daily mean temperature; RH, relative humidity; T_max_, daily maximum temperature; T_min_, daily minimum temperature; DTR, diurnal temperature range; MFS, meteorological factor score; GDM, gestational diabetes mellitus. Meteorological factor extremes were defined by different percentiles (5th, 3rd, 1st and 95th, 97th, 99th) of meteorological factors. All models were adjusted for maternal age, gravidity, parity, season of conception and year of conception.
