# Supplementary material for: Associations between meteorological factors and pregnancy complications during different pregnancy trimesters: a multicenter retrospective study in eastern China
Source: PeerJ. 2025 Jun 27;13:e19621. doi: 10.7717/peerj.19621 (PMC12208105; doi:10.7717/peerj.19621)
Supplement: Supplemental Information 17 — GDM, gestational diabetes mellitus; RERI, relative risk owing to interaction; AP, proportion attributable; 95% CI, 95% confidence interval; NA, Not applicable; Tmean, daily mean temperature; RH, relative humidity; Tmax, daily maximum temperature; Tmin, daily minimum temperature; DTR, diurnal temperature range. Extreme meteorological factors were defined by different percentiles (5th, 3rd, 1st and 95th, 97th, 99th) of meteorological factors. RERI and AP and their 95% confidence intervals are included. All models were adjusted for maternal age, gravidity, parity, season of conception and year of conception. [file peerj-13-19621-s017.docx]

**Supplemental Table S16** **Interaction between extreme meteorological factors on risks of GDM in different trimesters.**

| Gestational period | Meteorological factors | | RERI (95% *CI*) | AP (95% *CI*) |
| --- | --- | --- | --- | --- |
| The first trimester | Extreme low RH  (defined 5th percentile of RH) | Extreme high wind speed  (defined 95th percentile of wind speed) | 0.12 [-0.10, 0.35] | 0.12 [-0.13, 0.29] |
|  | Extreme low RH  (defined 5th percentile of RH) | Extreme high DTR  (defined 95th percentile of DTR) | 0.06 [-0.13, 0.26] | 0.06 [-0.16, 0.21] |
|  | Extreme high wind speed  (defined 95th percentile of wind speed) | Extreme high DTR  (defined 95th percentile of DTR) | 0.10 [-0.10, 0.31] | 0.11 [-0.13, 0.28] |
|  | Extreme low T_mean_  (defined 1st percentile of T_mean_) | Extreme high sunshine duration  (defined 99th percentile of sunshine duration) | NA [NA, NA] | NA [NA, NA] |
|  | Extreme low T_max_  (defined 1st percentile of T_max_) | Extreme high sunshine duration  (defined 99th percentile of sunshine duration) | NA [NA, NA] | NA [NA, NA] |
|  | Extreme low T_min_  (defined 1st percentile of T_min_) | Extreme high sunshine duration  (defined 99th percentile of sunshine duration) | NA [NA, NA] | NA [NA, NA] |
| The second trimester | Extreme low RH  (defined 5th percentile of RH) | Extreme low DTR  (defined 5th percentile of DTR) | NA [NA, NA] | NA [NA, NA] |
|  | Extreme low RH  (defined 5th percentile of RH) | Extreme high T_mean_  (defined 95th percentile of T_mean_) | NA [NA, NA] | NA [NA, NA] |
|  | Extreme low DTR  (defined 5th percentile of DTR) | Extreme high T_mean_  (defined 95th percentile of T_mean_) | NA [NA, NA] | NA [NA, NA] |
|  | Extreme low T_max_  (defined 3rd percentile of T_max_) | Extreme high wind speed  (defined 97th percentile of wind speed) | -0.11 [-0.44, 0.33] | -0.13 [-0.88, 0.11] |
|  | Extreme low T_min_  (defined 3rd percentile of T_min_) | Extreme high wind speed  (defined 97th percentile of wind speed) | -0.09 [-0.33, 0.15] | -0.10 [-0.44, 0.13] |
|  | Extreme low wind speed  (defined 1st percentile of wind speed) | Extreme low sunshine duration  (defined 1st percentile of sunshine duration) | NA [NA, NA] | NA [NA, NA] |
|  | Extreme low wind speed  (defined 1st percentile of wind speed) | Extreme high precipitation  (defined 99th percentile of precipitation) | NA [NA, NA] | NA [NA, NA] |
|  | Extreme low sunshine duration  (defined 1st percentile of sunshine duration) | Extreme high precipitation  (defined 99th percentile of precipitation) | NA [NA, NA] | NA [NA, NA] |
| The first two trimesters | Extreme low sunshine duration  (defined 5th percentile of sunshine duration) | Extreme low T_max_  (defined 5th percentile of T_max_) | 0.04 [-0.16, 0.24] | 0.04 [-0.19, 0.22] |
|  | Extreme low sunshine duration  (defined 5th percentile of sunshine duration) | Extreme low T_min_  (defined 5th percentile of T_min_) | NA [NA, NA] | NA [NA, NA] |
|  | Extreme low surface pressure  (defined 3rd percentile of surface pressure) | Extreme high T_mean_  (defined 97th percentile of T_mean_) | NA [NA, NA] | NA [NA, NA] |
|  | Extreme low surface pressure  (defined 3rd percentile of surface pressure) | Extreme high T_min_  (defined 97th percentile of T_min_) | -0.12 [-0.36, 0.11] | -0.12 [-0.40, 0.08] |
|  | Extreme low surface pressure  (defined 3rd percentile of surface pressure) | Extreme high precipitation  (defined 97th percentile of precipitation) | -0.13 [-0.39, 0.11] | -0.13 [-0.40, 0.09] |
|  | Extreme low surface pressure  (defined 3rd percentile of surface pressure) | Extreme high sunshine duration  (defined 97th percentile of sunshine duration) | -0.01 [-0.31, 0.36] | -0.01 [-0.50, 0.20] |
|  | Extreme high precipitation  (defined 97th percentile of precipitation) | Extreme high sunshine duration  (defined 97th percentile of sunshine duration) | NA [NA, NA] | NA [NA, NA] |
|  | Extreme high T_mean_  (defined 97th percentile of T_mean_) | Extreme high precipitation  (defined 97th percentile of precipitation) | NA [NA, NA] | NA [NA, NA] |
|  | Extreme high T_mean_  (defined 97th percentile of T_mean_) | Extreme high sunshine duration  (defined 97th percentile of sunshine duration) | 0.15 [-0.09, 0.39] | 0.14 [-0.12, 0.31] |
|  | Extreme high T_min_  (defined 97th percentile of T_min_) | Extreme high precipitation  (defined 97th percentile of precipitation) | -0.20 [-0.44, 0.04] | -0.19 [-0.48, 0.02] |
|  | Extreme high T_min_  (defined 97th percentile of T_min_) | Extreme high sunshine duration  (defined 97th percentile of sunshine duration) | 0.04 [-0.50, 1.10] | 0.04 [-1.47, 0.15] |
|  | Extreme low wind speed  (defined 1st percentile of wind speed) | Extreme low T_min_  (defined 1st percentile of T_min_) | NA [NA, NA] | NA [NA, NA] |

GDM, gestational diabetes mellitus; RERI, relative risk owing to interaction; AP, proportion attributable; 95% *CI*, 95% confidence interval; NA, Not applicable; T_mean_, daily mean temperature; RH, relative humidity; T_max_, daily maximum temperature; T_min_, daily minimum temperature; DTR, diurnal temperature range.

Extreme meteorological factors were defined by different percentiles (5th, 3rd, 1st and 95th, 97th, 99th) of meteorological factors. RERI and AP and their 95% confidence intervals are included. All models were adjusted for maternal age, gravidity, parity, season of conception and year of conception.
