# Supplementary material for: Associations between meteorological factors and pregnancy complications during different pregnancy trimesters: a multicenter retrospective study in eastern China
Source: PeerJ. 2025 Jun 27;13:e19621. doi: 10.7717/peerj.19621 (PMC12208105; doi:10.7717/peerj.19621)
Supplement: Supplemental Information 18 — GH, gestational hypertension; RERI, relative risk owing to interaction; AP, proportion attributable; 95% CI, 95% confidence interval; NA, Not applicable; Tmean, daily mean temperature; RH, relative humidity; Tmax, daily maximum temperature; Tmin, daily minimum temperature; DTR, diurnal temperature range. Extreme meteorological factors were defined by different percentiles (5th, 3rd, 1st and 95th, 97th, 99th) of meteorological factors. RERI and AP and their 95% confidence intervals are included. All models were adjusted for maternal age, gravidity, parity, season of conception and year of conception. [file peerj-13-19621-s018.docx]

**Supplemental Table S17 Interaction between extreme meteorological factors on risks of GH in different trimesters.**

| Gestational period | Meteorological factors | | RERI (95% *CI*) | AP (95% *CI*) |
| --- | --- | --- | --- | --- |
| The first trimester | Extreme low T_mean_  (defined 5th percentile of T_mean_) | Extreme low surface pressure  (defined 5th percentile of surface pressure) | NA [NA, NA] | NA [NA, NA] |
|  | Extreme low T_mean_  (defined 5th percentile of T_mean_) | Extreme high surface pressure  (defined 95th percentile of surface pressure) | 0.04 [-0.35, 0.40] | 0.04 [-0.47, 0.34] |
|  | Extreme high T_mean_  (defined 95th percentile of T_mean_) | Extreme low surface pressure  (defined 5th percentile of surface pressure) | -0.01 [-0.37, 0.35] | -0.01 [-0.44, 0.25] |
|  | Extreme high T_mean_  (defined 95th percentile of T_mean_) | Extreme high surface pressure  (defined 95th percentile of surface pressure) | NA [NA, NA] | NA [NA, NA] |
|  | Extreme low T_mean_  (defined 5th percentile of T_mean_) | Extreme low sunshine duration  (defined 5th percentile of sunshine duration) | -0.82 [-1.08, 1.11e+61] | -6641.28 [-1.33e+69, 7.31e+68] |
|  | Extreme high T_mean_  (defined 95th percentile of T_mean_) | Extreme low sunshine duration  (defined 5th percentile of sunshine duration) | NA [NA, NA] | NA [NA, NA] |
|  | Extreme low T_mean_  (defined 5th percentile of T_mean_) | Extreme high wind speed  (defined 95th percentile of wind speed) | -0.23 [-0.63, 0.18] | -0.26 [-0.96, 0.07] |
|  | Extreme high T_mean_  (defined 95th percentile of T_mean_) | Extreme high wind speed  (defined 95th percentile of wind speed) | NA [NA, NA] | NA [NA, NA] |
|  | Extreme low T_max_  (defined 5th percentile of T_max_) | Extreme low surface pressure  (defined 5th percentile of surface pressure) | NA [NA, NA] | NA [NA, NA] |
|  | Extreme low T_max_  (defined 5th percentile of T_max_) | Extreme high surface pressure  (defined 95th percentile of surface pressure) | -0.08 [-0.49, 0.30] | -0.08 [-0.64, 0.24] |
|  | Extreme low T_max_  (defined 5th percentile of T_max_) | Extreme low sunshine duration  (defined 5th percentile of sunshine duration) | -0.06 [-0.45, 0.37] | -0.07 [-0.82, 0.24] |
|  | Extreme low T_max_  (defined 5th percentile of T_max_) | Extreme high wind speed  (defined 95th percentile of wind speed) | -0.47 [-0.87, 0.02] | -0.61 [-1.90, -0.21] |
|  | Extreme low T_min_  (defined 5th percentile of T_min_) | Extreme low surface pressure  (defined 5th percentile of surface pressure) | NA [NA, NA] | NA [NA, NA] |
|  | Extreme low T_min_  (defined 5th percentile of T_min_) | Extreme high surface pressure  (defined 95th percentile of surface pressure) | -0.01 [-0.44, 0.36] | -0.01 [-0.58, 0.34] |
|  | Extreme high T_min_  (defined 95th percentile of T_min_) | Extreme low surface pressure  (defined 5th percentile of surface pressure) | -0.39 [-0.76, -0.05] | -0.44 [-0.98, -0.09] |
|  | Extreme high T_min_  (defined 95th percentile of T_min_) | Extreme high surface pressure  (defined 95th percentile of surface pressure) | NA [NA, NA] | NA [NA, NA] |
|  | Extreme low T_min_  (defined 5th percentile of T_min_) | Extreme low sunshine duration  (defined 5th percentile of sunshine duration) | NA [NA, NA] | NA [NA, NA] |
|  | Extreme high T_min_  (defined 95th percentile of T_min_) | Extreme low sunshine duration  (defined 5th percentile of sunshine duration) | NA [NA, NA] | NA [NA, NA] |
|  | Extreme low T_min_  (defined 5th percentile of T_min_) | Extreme high wind speed  (defined 95th percentile of wind speed) | -0.30 [-0.70, 0.08] | -0.34 [-0.99, 0.02] |
|  | Extreme high T_min_  (defined 95th percentile of T_min_) | Extreme high wind speed  (defined 95th percentile of wind speed) | NA [NA, NA] | NA [NA, NA] |
|  | Extreme low surface pressure  (defined 5th percentile of surface pressure) | Extreme low sunshine duration  (defined 5th percentile of sunshine duration) | NA [NA, NA] | NA [NA, NA] |
|  | Extreme high surface pressure  (defined 95th percentile of surface pressure) | Extreme low sunshine duration  (defined 5th percentile of sunshine duration) | -0.08 [-0.46, 0.35] | -0.11 [-1.06, 0.21] |
|  | Extreme low surface pressure  (defined 5th percentile of surface pressure) | Extreme high wind speed  (defined 95th percentile of wind speed) | NA [NA, NA] | NA [NA, NA] |
|  | Extreme high surface pressure  (defined 95th percentile of surface pressure) | Extreme high wind speed  (defined 95th percentile of wind speed) | -0.09 [-0.50, 0.27] | -0.1 [-0.62, 0.23] |
|  | Extreme low wind speed  (defined 3th percentile of wind speed) | Extreme low sunshine duration  (defined 3rd percentile of sunshine duration) | NA [NA, NA] | NA [NA, NA] |
|  | Extreme low wind speed  (defined 3th percentile of wind speed) | Extreme high precipitation  (defined 97th percentile of wind speed) | -0.28 [-0.80, 0.71] | -0.38 [-3.18, -0.04] |
|  | Extreme low sunshine duration  (defined 3rd percentile of sunshine duration) | Extreme high precipitation  (defined 97th percentile of wind speed) | NA [NA, NA] | NA [NA, NA] |
| The second trimester | Extreme low precipitation  (defined 5th percentile of precipitation) | Extreme low sunshine duration  (defined 5th percentile of sunshine duration) | NA [NA, NA] | NA [NA, NA] |
| The first two trimesters | Extreme low precipitation  (defined 5th percentile of precipitation) | Extreme low T_max_  (defined 5th percentile of T_max_) | -0.12 [-0.74, 0.96] | -0.11 [-1.67, 0.12] |

GH, gestational hypertension; RERI, relative risk owing to interaction; AP, proportion attributable; 95% *CI*, 95% confidence interval; NA, Not applicable; T_mean_, daily mean temperature; RH, relative humidity; T_max_, daily maximum temperature; T_min_, daily minimum temperature; DTR, diurnal temperature range.

Extreme meteorological factors were defined by different percentiles (5th, 3rd, 1st and 95th, 97th, 99th) of meteorological factors. RERI and AP and their 95% confidence intervals are included. All models were adjusted for maternal age, gravidity, parity, season of conception and year of conception.
