# Supplementary material for: Associations between meteorological factors and pregnancy complications during different pregnancy trimesters: a multicenter retrospective study in eastern China
Source: PeerJ. 2025 Jun 27;13:e19621. doi: 10.7717/peerj.19621 (PMC12208105; doi:10.7717/peerj.19621)
Supplement: Supplemental Information 19 — PE, preeclampsia; RERI, relative risk owing to interaction; AP, proportion attributable; 95% CI, 95% confidence interval; NA, Not applicable; Tmean, daily mean temperature; RH, relative humidity; Tmax, daily maximum temperature; Tmin, daily minimum temperature; DTR, diurnal temperature range. Extreme meteorological factors were defined by different percentiles (5th, 3rd, 1st and 95th, 97th, 99th) of meteorological factors. RERI and AP and their 95% confidence intervals are included. All models were adjusted for maternal age, gravidity, parity, season of conception and year of conception. [file peerj-13-19621-s019.docx]

**Supplemental Table S18 Interaction between extreme meteorological factors on risks of PE in different trimesters.**

| Gestational period | Meteorological factors | | RERI (95% *CI*) | AP (95% *CI*) |
| --- | --- | --- | --- | --- |
| The first trimester | Extreme low DTR  (defined 5th percentile of DTR) | Extreme high RH  (defined 95th percentile of RH) | 0.24 [-0.30, 1.06] | 0.20 [-0.59, 0.39] |
|  | Extreme low T_mean_  (defined 3rd percentile of T_max_) | Extreme low RH  (defined 3rd percentile of RH) | 0.22 [-0.34, 0.88] | 0.18 [-0.51, 0.44] |
|  | Extreme low T_mean_  (defined 3rd percentile of T_max_) | Extreme high RH  (defined 97th percentile of RH) | NA [NA, NA] | NA [NA, NA] |
|  | Extreme low T_mean_  (defined 3rd percentile of T_max_) | Extreme high DTR  (defined 97th percentile of DTR) | -0.27 [-0.86, 1.09] | -0.35 [-4.27, 0.33] |
|  | Extreme low RH  (defined 3rd percentile of RH) | Extreme high T_max_  (defined 97th percentile of T_max_) | NA [NA, NA] | NA [NA, NA] |
|  | Extreme low RH  (defined 3rd percentile of RH) | Extreme high T_min_  (defined 97th percentile of T_min_) | NA [NA, NA] | NA [NA, NA] |
|  | Extreme low RH  (defined 3rd percentile of RH) | Extreme high DTR  (defined 97th percentile of DTR) | -0.12 [-0.58, 0.35] | -0.15 [-1.08, 0.24] |
|  | Extreme high RH  (defined 97th percentile of RH) | Extreme high T_max_  (defined 97th percentile of T_max_) | NA [NA, NA] | NA [NA, NA] |
|  | Extreme high RH  (defined 97th percentile of RH) | Extreme high T_min_  (defined 97th percentile of T_min_) | -0.08 [-0.7, 1.07] | -0.08 [-2.02, 0.16] |
|  | Extreme high RH  (defined 97th percentile of RH) | Extreme high DTR  (defined 97th percentile of DTR) | NA [NA, NA] | NA [NA, NA] |
|  | Extreme high T_max_  (defined 97th percentile of T_max_) | Extreme high DTR  (defined 97th percentile of DTR) | -1.07 [-1.43, 9.97e+65] | -49650.5 [-4.46e+75, 2.16e+75] |
|  | Extreme high T_min_  (defined 97th percentile of T_min_) | Extreme high DTR  (defined 97th percentile of DTR) | NA [NA, NA] | NA [NA, NA] |
| The first two trimesters | Extreme low T_max_  (defined 5th percentile of T_max_) | Extreme high precipitation  (defined 95th percentile of precipitation) | NA [NA, NA] | NA [NA, NA] |
|  | Extreme low T_min_  (defined 1st percentile of T_min_) | Extreme high RH  (defined 99th percentile of RH) | NA [NA, NA] | NA [NA, NA] |
|  | Extreme low T_min_  (defined 1st percentile of T_min_) | Extreme high DTR  (defined 99th percentile of DTR) | 0.28 [-0.38, 0.94] | 0.44 [-1.29, 1.16] |
|  | Extreme high RH  (defined 99th percentile of RH) | Extreme high DTR  (defined 99th percentile of DTR) | NA [NA, NA] | NA [NA, NA] |

PE, preeclampsia; RERI, relative risk owing to interaction; AP, proportion attributable; 95% *CI*, 95% confidence interval; NA, Not applicable; T_mean_, daily mean temperature; RH, relative humidity; T_max_, daily maximum temperature; T_min_, daily minimum temperature; DTR, diurnal temperature range.

Extreme meteorological factors were defined by different percentiles (5th, 3rd, 1st and 95th, 97th, 99th) of meteorological factors. RERI and AP and their 95% confidence intervals are included. All models were adjusted for maternal age, gravidity, parity, season of conception and year of conception.
