# Supplementary material for: Associations between meteorological factors and pregnancy complications during different pregnancy trimesters: a multicenter retrospective study in eastern China
Source: PeerJ. 2025 Jun 27;13:e19621. doi: 10.7717/peerj.19621 (PMC12208105; doi:10.7717/peerj.19621)
Supplement: Supplemental Information 20 — RERI, relative risk owing to interaction; AP, proportion attributable; 95% CI, 95% confidence interval; NA, Not applicable; Tmean, daily mean temperature; RH, relative humidity; Tmax, daily maximum temperature; Tmin, daily minimum temperature; DTR, diurnal temperature range. Extremes meteorological factors were defined by different percentiles (5th, 3rd, 1st and 95th, 97th, 99th) of meteorological factors. RERI and AP and their 95% confidence intervals are included. All models were adjusted for maternal age, gravidity, Parity, season of conception and year of conception. [file peerj-13-19621-s020.docx]

**Supplemental Table S19 Interaction between extreme meteorological factors on risks of hypothyroidism in different trimesters.**

| Gestational period | Meteorological factors | | RERI (95% *CI*) | AP (95% *CI)* |
| --- | --- | --- | --- | --- |
| The first trimester | Extreme low RH  (defined 5th percentile of RH) | Extreme low wind speed  (defined 5th percentile of wind speed) | NA [NA, NA] | NA [NA, NA] |
|  | Extreme low RH  (defined 5th percentile of RH) | Extreme low precipitation  (defined 5th percentile of precipitation) | 0.04 [-0.16, 0.24] | 0.03 [-0.16, 0.18] |
|  | Extreme low RH  (defined 5th percentile of RH) | Extreme low sunshine duration  (defined 5th percentile of sunshine duration) | NA [NA, NA] | NA [NA, NA] |
|  | Extreme low RH  (defined 5th percentile of RH) | Extreme low T_min_  (defined 5th percentile of T_min_) | **0.25 [0.01, 0.49]** | **0.18 [0, 0.32]** |
|  | Extreme low RH  (defined 5th percentile of RH) | Extreme low DTR  (defined 5th percentile of DTR) | NA [NA, NA] | NA [NA, NA] |
|  | Extreme low RH  (defined 5th percentile of RH) | Extreme high T_mean_  (defined 95th percentile of T_mean_) | NA [NA, NA] | NA [NA, NA] |
|  | Extreme low RH  (defined 5th percentile of RH) | Extreme high wind speed  (defined 95th percentile of wind speed) | 0.18 [-0.18, 0.57] | 0.11 [-0.13, 0.28] |
|  | Extreme low RH  (defined 5th percentile of RH) | Extreme high sunshine duration  (defined 95th percentile of sunshine duration) | NA [NA, NA] | NA [NA, NA] |
|  | Extreme low RH  (defined 5th percentile of RH) | Extreme high T_max_  (defined 95th percentile of T_max_) | NA [NA, NA] | NA [NA, NA] |
|  | Extreme low RH  (defined 5th percentile of RH) | Extreme high T_min_  (defined 95th percentile of T_min_) | NA [NA, NA] | NA [NA, NA] |
|  | Extreme low RH  (defined 5th percentile of RH) | Extreme high DTR  (defined 95th percentile of DTR) | 0.19 [-0.03, 0.43] | 0.16 [-0.04, 0.30] |
|  | Extreme low wind speed  (defined 5th percentile of wind speed) | Extreme low precipitation  (defined 5th percentile of precipitation) | 0.03 [-0.23, 0.37] | 0.04 [-0.51, 0.22] |
|  | Extreme low wind speed  (defined 5th percentile of wind speed) | Extreme low sunshine duration  (defined 5th percentile of sunshine duration) | NA [NA, NA] | NA [NA, NA] |
|  | Extreme low wind speed  (defined 5th percentile of wind speed) | Extreme low T_min_  (defined 5th percentile of T_min_) | **-0.39 [-0.64, -0.07]** | **-0.64 [-1.74, -0.31]** |
|  | Extreme low wind speed  (defined 5th percentile of wind speed) | Extreme low DTR  (defined 5th percentile of DTR) | NA [NA, NA] | NA [NA, NA] |
|  | Extreme low wind speed  (defined 5th percentile of wind speed) | Extreme high T_mean_  (defined 95th percentile of T_mean_) | 0.11 [-0.22, 0.55] | 0.10 [-0.38, 0.28] |
|  | Extreme low wind speed  (defined 5th percentile of wind speed) | Extreme high RH  (defined 95th percentile of RH) | NA [NA, NA] | NA [NA, NA] |
|  | Extreme low wind speed  (defined 5th percentile of wind speed) | Extreme high sunshine duration  (defined 95th percentile of sunshine duration) | NA [NA, NA] | NA [NA, NA] |
|  | Extreme low wind speed  (defined 5th percentile of wind speed) | Extreme high T_max_  (defined 95th percentile of T_max_) | 0.17 [-0.15, 0.59] | 0.16 [-0.30, 0.32] |
|  | Extreme low wind speed  (defined 5th percentile of wind speed) | Extreme high T_min_  (defined 95th percentile of T_min_) | 0.20 [-0.12, 0.62] | 0.19 [-0.26, 0.34] |
|  | Extreme low wind speed  (defined 5th percentile of wind speed) | Extreme high DTR  (defined 95th percentile of DTR) | NA [NA, NA] | NA [NA, NA] |
|  | Extreme low precipitation  (defined 5th percentile of precipitation) | Extreme low sunshine duration  (defined 5th percentile of sunshine duration) | NA [NA, NA] | NA [NA, NA] |
|  | Extreme low precipitation  (defined 5th percentile of precipitation) | Extreme low T_min_  (defined 5th percentile of T_min_) | **0.60 [0.23, 1.03]** | **0.33 [0.13, 0.45]** |
|  | Extreme low precipitation  (defined 5th percentile of precipitation) | Extreme low DTR  (defined 5th percentile of DTR) | NA [NA, NA] | NA [NA, NA] |
|  | Extreme low precipitation  (defined 5th percentile of precipitation) | Extreme high T_mean_  (defined 95th percentile of T_mean_) | NA [NA, NA] | NA [NA, NA] |
|  | Extreme low precipitation  (defined 5th percentile of precipitation) | Extreme high RH  (defined 95th percentile of RH) | NA [NA, NA] | NA [NA, NA] |
|  | Extreme low precipitation  (defined 5th percentile of precipitation) | Extreme high wind speed  (defined 95th percentile of wind speed) | **0.43 [0.06, 0.79]** | **0.25 [0.03, 0.41]** |
|  | Extreme low precipitation  (defined 5th percentile of precipitation) | Extreme high sunshine duration  (defined 95th percentile of sunshine duration) | NA [NA, NA] | NA [NA, NA] |
|  | Extreme low precipitation  (defined 5th percentile of precipitation) | Extreme high T_max_  (defined 95th percentile of T_max_) | NA [NA, NA] | NA [NA, NA] |
|  | Extreme low precipitation  (defined 5th percentile of precipitation) | Extreme high T_min_  (defined 95th percentile of T_min_) | NA [NA, NA] | NA [NA, NA] |
|  | Extreme low precipitation  (defined 5th percentile of precipitation) | Extreme high DTR  (defined 95th percentile of DTR) | **0.63 [0.33, 0.97]** | **0.40 [0.23, 0.51]** |
|  | Extreme low sunshine duration  (defined 5th percentile of sunshine duration) | Extreme low T_min_  (defined 5th percentile of T_min_) | NA [NA, NA] | NA [NA, NA] |
|  | Extreme low sunshine duration  (defined 5th percentile of sunshine duration) | Extreme low DTR  (defined 5th percentile of DTR) | **0.40 [0.23, 0.57]** | **0.37 [0.21, 0.49]** |
|  | Extreme low sunshine duration  (defined 5th percentile of sunshine duration) | Extreme high T_mean_  (defined 95th percentile of T_mean_) | NA [NA, NA] | NA [NA, NA] |
|  | Extreme low sunshine duration  (defined 5th percentile of sunshine duration) | Extreme high RH  (defined 95th percentile of RH) | NA [NA, NA] | NA [NA, NA] |
|  | Extreme low sunshine duration  (defined 5th percentile of sunshine duration) | Extreme high wind speed  (defined 95th percentile of wind speed) | NA [NA, NA] | NA [NA, NA] |
|  | Extreme low sunshine duration  (defined 5th percentile of sunshine duration) | Extreme high T_max_  (defined 95th percentile of T_max_) | NA [NA, NA] | NA [NA, NA] |
|  | Extreme low sunshine duration  (defined 5th percentile of sunshine duration) | Extreme high T_min_  (defined 95th percentile of T_min_) | NA [NA, NA] | NA [NA, NA] |
|  | Extreme low sunshine duration  (defined 5th percentile of sunshine duration) | Extreme high DTR  (defined 95th percentile of DTR) | NA [NA, NA] | NA [NA, NA] |
|  | Extreme low T_min_  (defined 5th percentile of T_min_) | Extreme low DTR  (defined 5th percentile of DTR) | NA [NA, NA] | NA [NA, NA] |
|  |  | Extreme high RH  (defined 95th percentile of RH) | NA [NA, NA] | NA [NA, NA] |
|  | Extreme low T_min_  (defined 5th percentile of T_min_) | Extreme high wind speed  (defined 95th percentile of wind speed) | **1.06 [0.60, 1.61]** | **0.44 [0.27, 0.54]** |
|  | Extreme low T_min_  (defined 5th percentile of T_min_) | Extreme high sunshine duration  (defined 95th percentile of sunshine duration) | NA [NA, NA] | NA [NA, NA] |
|  | Extreme low T_min_  (defined 5th percentile of T_min_) | Extreme high DTR  (defined 95th percentile of DTR) | **0.74 [0.33, 1.22]** | **0.39 [0.19, 0.50]** |
|  | Extreme low DTR  (defined 5th percentile of DTR) | Extreme high T_mean_  (defined 95th percentile of T_mean_) | NA [NA, NA] | NA [NA, NA] |
|  | Extreme low DTR  (defined 5th percentile of DTR) | Extreme high RH  (defined 95th percentile of RH) | -0.24 [-0.57, 0.21] | -0.22 [-0.81, 0.04] |
|  | Extreme low DTR  (defined 5th percentile of DTR) | Extreme high wind speed  (defined 95th percentile of wind speed) | NA [NA, NA] | NA [NA, NA] |
|  | Extreme low DTR  (defined 5th percentile of DTR) | Extreme high sunshine duration  (defined 95th percentile of sunshine duration) | NA [NA, NA] | NA [NA, NA] |
|  | Extreme low DTR  (defined 5th percentile of DTR) | Extreme high T_max_  (defined 95th percentile of T_max_) | NA [NA, NA] | NA [NA, NA] |
|  | Extreme low DTR  (defined 5th percentile of DTR) | Extreme high T_min_  (defined 95th percentile of T_min_) | NA [NA, NA] | NA [NA, NA] |
|  | Extreme high T_mean_  (defined 95th percentile of T_mean_) | Extreme high RH  (defined 95th percentile of RH) | NA [NA, NA] | NA [NA, NA] |
|  | Extreme high T_mean_  (defined 95th percentile of T_mean_) | Extreme high wind speed  (defined 95th percentile of wind speed) | NA [NA, NA] | NA [NA, NA] |
|  | Extreme high T_mean_  (defined 95th percentile of T_mean_) | Extreme high sunshine duration  (defined 95th percentile of sunshine duration) | **-0.35 [-0.61, -0.11]** | **-0.33 [-0.62, -0.11]** |
|  | Extreme high T_mean_  (defined 95th percentile of T_mean_) | Extreme high DTR  (defined 95th percentile of DTR) | -0.25 [-0.66, 0.39] | **-0.27 [-1.45, -0.03]** |
|  | Extreme high RH  (defined 95th percentile of RH) | Extreme high wind speed  (defined 95th percentile of wind speed) | NA [NA, NA] | NA [NA, NA] |
|  | Extreme high RH  (defined 95th percentile of RH) | Extreme high sunshine duration  (defined 95th percentile of sunshine duration) | NA [NA, NA] | NA [NA, NA] |
|  | Extreme high RH  (defined 95th percentile of RH) | Extreme high T_max_  (defined 95th percentile of T_max_) | NA [NA, NA] | NA [NA, NA] |
|  | Extreme high RH  (defined 95th percentile of RH) | Extreme high T_min_  (defined 95th percentile of T_min_) | **-0.39 [-0.63, -0.13]** | **-0.39 [-0.78, -0.15]** |
|  | Extreme high RH  (defined 95th percentile of RH) | Extreme high DTR  (defined 95th percentile of DTR) | NA [NA, NA] | NA [NA, NA] |
|  | Extreme high wind speed  (defined 95th percentile of wind speed) | Extreme high sunshine duration  (defined 95th percentile of sunshine duration) | NA [NA, NA] | NA [NA, NA] |
|  | Extreme high wind speed  (defined 95th percentile of wind speed) | Extreme high T_max_  (defined 95th percentile of T_max_) | NA [NA, NA] | NA [NA, NA] |
|  | Extreme high wind speed  (defined 95th percentile of wind speed) | Extreme high T_min_  (defined 95th percentile of T_min_) | NA [NA, NA] | NA [NA, NA] |
|  | Extreme high wind speed  (defined 95th percentile of wind speed) | Extreme high DTR  (defined 95th percentile of DTR) | **0.66 [0.29, 1.05]** | **0.36 [0.17, 0.49]** |
|  | Extreme high sunshine duration  (defined 95th percentile of sunshine duration) | Extreme high T_max_  (defined 95th percentile of T_max_) | **-0.34 [-0.57, -0.12]** | **-0.39 [-0.73, -0.14]** |
|  | Extreme high sunshine duration  (defined 95th percentile of sunshine duration) | Extreme high T_min_  (defined 95th percentile of T_min_) | -0.14 [-0.36, 0.06] | -0.14 [-0.39, 0.05] |
|  | Extreme high sunshine duration  (defined 95th percentile of sunshine duration) | Extreme high DTR  (defined 95th percentile of DTR) | -0.24 [-0.61, 0.33] | **-0.28 [-1.42, -0.04]** |
|  | Extreme high T_max_  (defined 95th percentile of T_max_) | Extreme high DTR  (defined 95th percentile of DTR) | -0.21 [-0.58, 0.37] | **-0.24 [-1.36, -0.02]** |
|  | Extreme high T_min_  (defined 95th percentile of T_min_) | Extreme high DTR  (defined 95th percentile of DTR) | -0.06 [-0.48, 0.62] | -0.07 [-1.16, 0.10] |
|  | Extreme low T_mean_  (defined 3rd percentile of T_mean_) | Extreme low RH  (defined 3rd percentile of RH) | 0.05 [-0.25, 0.37] | 0.04 [-0.23, 0.23] |
|  | Extreme low T_mean_  (defined 3rd percentile of T_mean_) | Extreme low precipitation  (defined 3rd percentile of precipitation) | 0.52 [-0.50, 3.09] | 0.31 [-1.31, 0.47] |
|  | Extreme low T_mean_  (defined 3rd percentile of T_mean_) | Extreme low sunshine duration  (defined 3rd percentile of sunshine duration) | NA [NA, NA] | NA [NA, NA] |
|  | Extreme low T_mean_  (defined 3rd percentile of T_mean_) | Extreme low DTR  (defined 3rd percentile of DTR) | NA [NA, NA] | NA [NA, NA] |
|  | Extreme low T_mean_  (defined 3rd percentile of T_mean_) | Extreme high RH  (defined 97th percentile of RH) | NA [NA, NA] | NA [NA, NA] |
|  | Extreme low T_mean_  (defined 3rd percentile of T_mean_) | Extreme high wind speed  (defined 97th percentile of wind speed) | **3.02 [1.45, 5.36]** | **0.65 [0.42, 0.74]** |
|  | Extreme low T_mean_  (defined 3rd percentile of T_mean_) | Extreme high precipitation  (defined 97th percentile of precipitation) | NA [NA, NA] | NA [NA, NA] |
|  | Extreme low T_mean_  (defined 3rd percentile of T_mean_) | Extreme high sunshine duration  (defined 97th percentile of sunshine duration) | NA [NA, NA] | NA [NA, NA] |
|  | Extreme low T_mean_  (defined 3rd percentile of T_mean_) | Extreme high DTR  (defined 97th percentile of DTR) | **2.53 [0.69, 6.17]** | **0.67 [0.23, 0.72]** |
|  | Extreme low RH  (defined 3rd percentile of RH) | Extreme low precipitation  (defined 3rd percentile of precipitation) | 0.06 [-0.21, 0.33] | 0.04 [-0.20, 0.22] |
|  | Extreme low RH  (defined 3rd percentile of RH) | Extreme low sunshine duration  (defined 3rd percentile of sunshine duration) | NA [NA, NA] | NA [NA, NA] |
|  | Extreme low RH  (defined 3rd percentile of RH) | Extreme low DTR  (defined 3rd percentile of DTR) | NA [NA, NA] | NA [NA, NA] |
|  | Extreme low RH  (defined 3rd percentile of RH) | Extreme high wind speed  (defined 97th percentile of wind speed) | -0.13 [-0.78, 0.62] | -0.07 [-0.55, 0.20] |
|  | Extreme low RH  (defined 3rd percentile of RH) | Extreme high precipitation  (defined 97th percentile of precipitation) | NA [NA, NA] | NA [NA, NA] |
|  | Extreme low RH  (defined 3rd percentile of RH) | Extreme high sunshine duration  (defined 97th percentile of sunshine duration) | NA [NA, NA] | NA [NA, NA] |
|  | Extreme low RH  (defined 3rd percentile of RH) | Extreme high T_max_  (defined 97th percentile of T_max_) | NA [NA, NA] | NA [NA, NA] |
|  | Extreme low RH  (defined 3rd percentile of RH) | Extreme high T_min_  (defined 97th percentile of T_min_) | NA [NA, NA] | NA [NA, NA] |
|  | Extreme low RH  (defined 3rd percentile of RH) | Extreme high DTR  (defined 97th percentile of DTR) | -0.17 [-0.49, 0.18] | -0.14 [-0.49, 0.09] |
|  | Extreme low precipitation  (defined 3rd percentile of precipitation) | Extreme low sunshine duration  (defined 3rd percentile of sunshine duration) | NA [NA, NA] | NA [NA, NA] |
|  | Extreme low precipitation  (defined 3rd percentile of precipitation) | Extreme low DTR  (defined 3rd percentile of DTR) | NA [NA, NA] | NA [NA, NA] |
|  | Extreme low precipitation  (defined 3rd percentile of precipitation) | Extreme high RH  (defined 97th percentile of RH) | NA [NA, NA] | NA [NA, NA] |
|  | Extreme low precipitation  (defined 3rd percentile of precipitation) | Extreme high wind speed  (defined 97th percentile of wind speed) | **-0.73 [-1.45, -0.1]** | **-0.48 [-1.1, -0.08]** |
|  | Extreme low precipitation  (defined 3rd percentile of precipitation) | Extreme high sunshine duration  (defined 97th percentile of sunshine duration) | NA [NA, NA] | NA [NA, NA] |
|  | Extreme low precipitation  (defined 3rd percentile of precipitation) | Extreme high T_max_  (defined 97th percentile of T_max_) | NA [NA, NA] | NA [NA, NA] |
|  | Extreme low precipitation  (defined 3rd percentile of precipitation) | Extreme high T_min_  (defined 97th percentile of T_min_) | NA [NA, NA] | NA [NA, NA] |
|  | Extreme low precipitation  (defined 3rd percentile of precipitation) | Extreme high DTR  (defined 97th percentile of DTR) | **0.38 [0.04, 0.78]** | **0.27 [0, 0.43]** |
|  | Extreme low sunshine duration  (defined 3rd percentile of sunshine duration) | Extreme low DTR  (defined 3rd percentile of DTR) | **0.31 [0.10, 0.52]** | **0.30 [0.10, 0.45]** |
|  | Extreme low sunshine duration  (defined 3rd percentile of sunshine duration) | Extreme high RH  (defined 97th percentile of RH) | NA [NA, NA] | NA [NA, NA] |
|  | Extreme low sunshine duration  (defined 3rd percentile of sunshine duration) | Extreme high wind speed  (defined 97th percentile of wind speed) | NA [NA, NA] | NA [NA, NA] |
|  | Extreme low sunshine duration  (defined 3rd percentile of sunshine duration) | Extreme high precipitation  (defined 97th percentile of precipitation) | NA [NA, NA] | NA [NA, NA] |
|  | Extreme low sunshine duration  (defined 3rd percentile of sunshine duration) | Extreme high T_max_  (defined 97th percentile of T_max_) | NA [NA, NA] | NA [NA, NA] |
|  | Extreme low sunshine duration  (defined 3rd percentile of sunshine duration) | Extreme high T_min_  (defined 97th percentile of T_min_) | NA [NA, NA] | NA [NA, NA] |
|  | Extreme low sunshine duration  (defined 3rd percentile of sunshine duration) | Extreme high DTR  (defined 97th percentile of DTR) | NA [NA, NA] | NA [NA, NA] |
|  | Extreme low DTR  (defined 3rd percentile of DTR) | Extreme high RH  (defined 97th percentile of RH) | -0.23 [-0.60, 0.29] | -0.24 [-1.10, 0.03] |
|  | Extreme low DTR  (defined 3rd percentile of DTR) | Extreme high wind speed  (defined 97th percentile of wind speed) | NA [NA, NA] | NA [NA, NA] |
|  | Extreme low DTR  (defined 3rd percentile of DTR) | Extreme high precipitation  (defined 97th percentile of precipitation) | NA [NA, NA] | NA [NA, NA] |
|  | Extreme low DTR  (defined 3rd percentile of DTR) | Extreme high sunshine duration  (defined 97th percentile of sunshine duration) | NA [NA, NA] | NA [NA, NA] |
|  | Extreme low DTR  (defined 3rd percentile of DTR) | Extreme high T_max_  (defined 97th percentile of T_max_) | NA [NA, NA] | NA [NA, NA] |
|  | Extreme low DTR  (defined 3rd percentile of DTR) | Extreme high T_min_  (defined 97th percentile of T_min_) | NA [NA, NA] | NA [NA, NA] |
|  | Extreme high RH  (defined 97th percentile of RH) | Extreme high wind speed  (defined 97th percentile of wind speed) | NA [NA, NA] | NA [NA, NA] |
|  | Extreme high RH  (defined 97th percentile of RH) | Extreme high precipitation  (defined 97th percentile of precipitation) | 0.27 [-0.03, 0.54] | 0.20 [-0.03, 0.37] |
|  | Extreme high RH  (defined 97th percentile of RH) | Extreme high sunshine duration  (defined 97th percentile of sunshine duration) | NA [NA, NA] | NA [NA, NA] |
|  | Extreme high RH  (defined 97th percentile of RH) | Extreme high T_max_  (defined 97th percentile of T_max_) | NA [NA, NA] | NA [NA, NA] |
|  | Extreme high RH  (defined 97th percentile of RH) | Extreme high T_min_  (defined 97th percentile of T_min_) | -0.07 [-0.43, 0.39] | -0.05 [-0.53, 0.18] |
|  | Extreme high RH  (defined 97th percentile of RH) | Extreme high DTR  (defined 97th percentile of DTR) | NA [NA, NA] | NA [NA, NA] |
|  | Extreme high wind speed  (defined 97th percentile of wind speed) | Extreme high precipitation  (defined 97th percentile of precipitation) | NA [NA, NA] | NA [NA, NA] |
|  | Extreme high wind speed  (defined 97th percentile of wind speed) | Extreme high sunshine duration  (defined 97th percentile of sunshine duration) | NA [NA, NA] | NA [NA, NA] |
|  | Extreme high wind speed  (defined 97th percentile of wind speed) | Extreme high T_max_  (defined 97th percentile of T_max_) | NA [NA, NA] | NA [NA, NA] |
|  | Extreme high wind speed  (defined 97th percentile of wind speed) | Extreme high T_min_  (defined 97th percentile of T_min_) | NA [NA, NA] | NA [NA, NA] |
|  | Extreme high wind speed  (defined 97th percentile of wind speed) | Extreme high DTR  (defined 97th percentile of DTR) | 0.31 [-0.33, 1.04] | 0.15 [-0.22, 0.37] |
|  | Extreme high precipitation  (defined 97th percentile of precipitation) | Extreme high sunshine duration  (defined 97th percentile of sunshine duration) | NA [NA, NA] | NA [NA, NA] |
|  | Extreme high precipitation  (defined 97th percentile of precipitation) | Extreme high T_max_  (defined 97th percentile of T_max_) | NA [NA, NA] | NA [NA, NA] |
|  | Extreme high precipitation  (defined 97th percentile of precipitation) | Extreme high T_min_  (defined 97th percentile of T_min_) | -0.03 [-0.39, 0.43] | -0.02 [-0.48, 0.20] |
|  | Extreme high precipitation  (defined 97th percentile of precipitation) | Extreme high DTR  (defined 97th percentile of DTR) | NA [NA, NA] | NA [NA, NA] |
|  | Extreme high sunshine duration  (defined 97th percentile of sunshine duration) | Extreme high T_max_  (defined 97th percentile of T_max_) | **-0.29 [-0.6, 0]** | **-0.32 [-0.74, -0.02]** |
|  | Extreme high sunshine duration  (defined 97th percentile of sunshine duration) | Extreme high T_min_  (defined 97th percentile of T_min_) | -0.25 [-0.54, 0.03] | -0.25 [-0.61, 0.01] |
|  | Extreme high sunshine duration  (defined 97th percentile of sunshine duration) | Extreme high DTR  (defined 97th percentile of DTR) | -0.44 [-1.08, 4.01] | -0.63 [-18.73, 5.74] |
|  | Extreme high T_max_  (defined 97th percentile of T_max_) | Extreme high DTR  (defined 97th percentile of DTR) | -0.46 [-1.12, 4.10] | -0.65 [-18.68, 5.52] |
|  | Extreme high T_min_  (defined 97th percentile of T_min_) | Extreme high DTR  (defined 97th percentile of DTR) | NA [NA, NA] | NA [NA, NA] |
|  | Extreme low T_mean_  (defined 1st percentile of T_mean_) | Extreme low RH  (defined 1st percentile of RH) | -0.24 [-1.08, 1.69] | **-0.19 [-2.77, -0.01]** |
|  | Extreme low T_mean_  (defined 1st percentile of T_mean_) | Extreme low wind speed  (defined 1st percentile of wind speed) | NA [NA, NA] | NA [NA, NA] |
|  | Extreme low T_mean_  (defined 1st percentile of T_mean_) | Extreme high RH  (defined 99th percentile of RH) | NA [NA, NA] | NA [NA, NA] |
|  | Extreme low T_mean_  (defined 1st percentile of T_mean_) | Extreme high surface pressure  (defined 99th percentile of surface pressure) | **2.02 [0.27, 5.07]** | **0.54 [0.03, 0.68]** |
|  | Extreme low T_mean_  (defined 1st percentile of T_mean_) | Extreme high wind speed  (defined 99th percentile of wind speed) | -5.43 [-16.09, 6.81] | **-3.07 [-38.72, -1.58]** |
|  | Extreme low RH  (defined 1st percentile of RH) | Extreme low wind speed  (defined 1st percentile of wind speed) | NA [NA, NA] | NA [NA, NA] |
|  | Extreme low RH  (defined 1st percentile of RH) | Extreme low T_min_  (defined 1st percentile of T_min_) | **-1.66 [-2.69, -0.82]** | **-1.10 [-2.05, -0.50]** |
|  | Extreme low RH  (defined 1st percentile of RH) | Extreme high T_mean_  (defined 99th percentile of T_mean_) | NA [NA, NA] | NA [NA, NA] |
|  | Extreme low RH  (defined 1st percentile of RH) | Extreme high surface pressure  (defined 99th percentile of surface pressure) | **-1.09 [-2.2, -0.18]** | **-0.63 [-1.48, -0.12]** |
|  | Extreme low RH  (defined 1st percentile of RH) | Extreme high wind speed  (defined 99th percentile of wind speed) | NA [NA, NA] | NA [NA, NA] |
|  | Extreme low wind speed  (defined 1st percentile of wind speed) | Extreme low T_min_  (defined 1st percentile of T_min_) | NA [NA, NA] | NA [NA, NA] |
|  | Extreme low wind speed  (defined 1st percentile of wind speed) | Extreme high T_mean_  (defined 99th percentile of T_mean_) | NA [NA, NA] | NA [NA, NA] |
|  | Extreme low wind speed  (defined 1st percentile of wind speed) | Extreme high RH  (defined 99th percentile of RH) | NA [NA, NA] | NA [NA, NA] |
|  | Extreme low wind speed  (defined 1st percentile of wind speed) | Extreme high surface pressure  (defined 99th percentile of surface pressure) | NA [NA, NA] | NA [NA, NA] |
|  | Extreme low T_min_  (defined 1st percentile of T_min_) | Extreme high RH  (defined 99th percentile of RH) | NA [NA, NA] | NA [NA, NA] |
|  | Extreme low T_min_  (defined 1st percentile of T_min_) | Extreme high surface pressure  (defined 99th percentile of surface pressure) | 0.20 [-0.85, 1.50] | 0.07 [-0.48, 0.36] |
|  | Extreme low T_min_  (defined 1st percentile of T_min_) | Extreme high wind speed  (defined 99th percentile of wind speed) | 3.28 [-1.01e+106, 10.06] | 0.62 [-1.91e+105, 0.86] |
|  | Extreme high T_mean_  (defined 99th percentile of T_mean_) | Extreme high RH  (defined 99th percentile of RH) | NA [NA, NA] | NA [NA, NA] |
|  | Extreme high T_mean_  (defined 99th percentile of T_mean_) | Extreme high surface pressure  (defined 99th percentile of surface pressure) | NA [NA, NA] | NA [NA, NA] |
|  | Extreme high T_mean_  (defined 99th percentile of T_mean_) | Extreme high wind speed  (defined 99th percentile of wind speed) | NA [NA, NA] | NA [NA, NA] |
|  | Extreme high RH  (defined 99th percentile of RH) | Extreme high surface pressure  (defined 99th percentile of surface pressure) | NA [NA, NA] | NA [NA, NA] |
|  |  | Extreme high wind speed  (defined 99th percentile of wind speed) | NA [NA, NA] | NA [NA, NA] |
|  | Extreme high surface pressure  (defined 99th percentile of surface pressure) | Extreme high wind speed  (defined 99th percentile of wind speed) | -6.63 [-17.76, 6.02] | **-3.64 [-42.72, -1.98]** |

RERI, relative risk owing to interaction; AP, proportion attributable; 95% *CI*, 95% confidence interval; NA, Not applicable; T_mean_, daily mean temperature; RH, relative humidity; T_max_, daily maximum temperature; T_min_, daily minimum temperature; DTR, diurnal temperature range.

Extremes meteorological factors were defined by different percentiles (5th, 3rd, 1st and 95th, 97th, 99th) of meteorological factors. RERI and AP and their 95% confidence intervals are included. All models were adjusted for maternal age, gravidity, Parity, season of conception and year of conception.
