# Supplementary material for: Associations between meteorological factors and pregnancy complications during different pregnancy trimesters: a multicenter retrospective study in eastern China
Source: PeerJ. 2025 Jun 27;13:e19621. doi: 10.7717/peerj.19621 (PMC12208105; doi:10.7717/peerj.19621)
Supplement: Supplemental Information 23 [file peerj-13-19621-s023.pdf]

Categorical data codebook

Code for gravidity

Value	Meaning of Value	Description
1	Once	None
2	Twice	None
3	Greater than or equal to three times	None

Code of parity

Value	Meaning of Value	Description
1	Primiparous	None
2	Multiparous	None

Code of season of conception

Value	Meaning of Value	Description
1	Spring	March–May
2	Summer	June–August
3	Fall	September–November
4	Winter	December–February

Code of residence

Value	Meaning of Value	Description
1	Residents	None
2	Immigrants	None

Code of fetal gender

Value	Meaning of Value	Description
0	Missing	None
1	Male	None
2	Female	None

Code of GDM

Value	Meaning of Value	Description
0	Non-GDM	None
1	GDM	Gestational diabetes mellitus, GDM

Code of GH

Value	Meaning of Value	Description
0	Non-GH	None
1	GH	Gestational hypertension, GH

Code of PE

Value	Meaning of Value	Description
0	Non-PE	None
1	PE	Preeclampsia, PE

Code of hypothyroidism

Value	Meaning of Value	Description
0	Non-hypothyroidism	None
1	hypothyroidism	None
