# Supplementary material for: Associations between meteorological factors and pregnancy complications during different pregnancy trimesters: a multicenter retrospective study in eastern China
Source: PeerJ. 2025 Jun 27;13:e19621. doi: 10.7717/peerj.19621 (PMC12208105; doi:10.7717/peerj.19621)
Supplement: Supplemental Information 24 [file peerj-13-19621-s024.docx]

STROBE Statement—checklist of items that should be included in reports of observational studies

|  | Item No. | Recommendation | Page  No. | Relevant text from manuscript |
| --- | --- | --- | --- | --- |
| **Title and abstract** | 1 | (*a*) Indicate the study’s design with a commonly used term in the title or the abstract | 1 | Associations between meteorological factors and pregnancy complications during different pregnancy trimesters: A multicenter cross-sectional study in eastern China |
|  |  | (*b*) Provide in the abstract an informative and balanced summary of what was done and what was found | 2 | **Background.** Previous studies have demonstrated the effects of ambient temperature on the risk of pregnancy complications. However, the associations between multiple meteorological factors and pregnancy complications have rarely been studied.  **Methods.** We carried out a retrospective study on the impacts of meteorological factors on pregnancy complications in different trimesters in Ningbo city, China, from 2013–2023. Daily meteorological factors data were obtained from the National Aeronautics and Space Administration (NASA). Moreover, a meteorological factor score (MFS) was calculated. Logistic regression models were applied to assess the effects of individual meteorological factors and MFS on pregnancy complications during different trimesters. Distributed lag nonlinear models were used to explore the sensitive time windows of extreme meteorological factors in different weeks of gestation. And the interaction effects of extreme meteorological factors were assessed.  **Results.** A total of 92,332 participants were included; 17,814 (19.29%) were diagnosed with gestational diabetes mellitus (GDM), 3860 (4.18%) were diagnosed with gestational hypertension (GH), 3101 (3.36%) were diagnosed with preeclampsia (PE), and 17,418 (18.86%) were diagnosed with hypothyroidism. Compared with participants with MFS in the lowest quintile, participants with MFS in higher quintile groups had greater risks of GDM (aOR = 1.186, 95% CI: 1.079–1.304), GH (aOR = 1.596, 95% CI: 1.323–1.925), PE (aOR = 1.347, 95% CI: 1.094–1.658) and hypothyroidism (aOR = 1.257, 95% CI: 1.141–1.385) in the first trimester. Extremely high and low meteorological factors, especially during the 9th to 22nd gestational weeks, were associated with increased risks of GDM, GH and PE, whereas extremely high and low meteorological factors during the 1st to 13th gestational weeks were associated with an increased risk of hypothyroidism. Interactions between extreme meteorological factors on hypothyroidism were observed.  **Conclusions.** Our finding showed that meteorological factors were critical risk factors for pregnancy complications. Targeted protective measures should be taken to reduce the risk of pregnancy complications. |
| Introduction | | | |  |
| Background/rationale | 2 | Explain the scientific background and rationale for the investigation being reported | 2-3 | In recent years, global meteorological factors have changed dramatically, which have been the biggest global health threat (Costello et al., 2009). Meteorological factors are comprehensive factors that include temperature, humidity, air pressure, precipitation, and wind speed. The impact of meteorological factors on health is also of increasing concern (Romanello et al., 2023). Complex meteorological factors increase the population health risk, including metabolic disorders, cardiorespiratory diseases, reproductive health and infectious diseases (Chen et al., 2018; Andhikaputra et al., 2023; Ratter-Rieck, Roden & Herder, 2023). Climate change may lead to increases in the frequency, intensity and duration of extreme temperatures globally. High temperatures may lead to heat-related illnesses, increase the burden on the heart and exacerbate the symptoms of cardiovascular disease. Conversely, low temperatures may lead to hypothermia, which affects the cardiovascular system and increases the risk of heart attack. Extreme temperatures also affect reproductive processes such as spermatogenesis, ovarian reserve and placental development (Romanello et al., 2023). Climate change can exacerbate air pollution and increase the concentrations of particulate matter and ozone, which can affect the health of the respiratory and cardiovascular systems and reproductive health (Segal & Giudice, 2022; Liang et al., 2023). In addition, climate change affects hormone levels and the immune response and interferes with the normal regulation of the endocrine system due to intensifying temperature changes, air pollution, ultraviolet radiation and psychological stress (Yüzen et al., 2023; Hannan et al., 2024).  Recent studies have indicated that complex meteorological factors also affect the health of pregnant women and are closely related to the occurrence of several pregnancy complications, such as gestational diabetes mellitus (GDM), gestational hypertension (GH), preeclampsia (PE), and hypothyroidism (Yan et al., 2022). A study in Toronto reported that an increased risk of GDM was associated with each 10 °C increase in the mean 30-day outdoor average temperature before GDM screening (Booth et al., 2017). A study in Guangzhou, China, revealed that during specific gestational weeks in the second trimester, both extremely high and extremely low temperatures and the diurnal temperature range (DTR) could increase the risk of GDM (Zhang et al., 2021). A review demonstrated that heat exposure increased the risk of PE and GH, whereas cold exposure decreased the risk (Mao et al., 2023). Seasonal changes could affect the incidence of GDM and hypertensive disorders of pregnancy (HDP) (Chiefari et al., 2017; Rohr Thomsen et al., 2020). A multicenter study reported the associations between solar radiation and HDP (Lu et al., 2022).  Previous studies have largely emphasized the impact of extreme temperature, a single meteorological factor, on pregnancy complications, but few studies have analyzed the effects of other meteorological factors, such as humidity, air pressure, wind speed, precipitation and sunshine duration. There is an even greater lack of comprehensive evaluations of the cumulative and interactive effects of meteorological factors on pregnancy complications. |
| Objectives | 3 | State specific objectives, including any prespecified hypotheses | 3 | This study was based on a multicenter cross-sectional study in eastern China that included 92,332 people. In this study, we aimed to comprehensively evaluate the independent risk effects of different meteorological factors on pregnancy complications and construct a comprehensive meteorological factor score (MFS) to analyze the correlation between the overall exposure level of meteorological factors and disease. These results could play a positive role in the early prevention of pregnancy complications in a time of increasing climate change. |
| Methods | | | |  |
| Study design | 4 | Present key elements of study design early in the paper | 3-7 | This study is based on a multicenter retrospective cross-sectional study conducted in Ningbo city, China (Zhu et al., 2024a,b). |
| Setting | 5 | Describe the setting, locations, and relevant dates, including periods of recruitment, exposure, follow-up, and data collection | 3-4 | Ningbo city, located on the eastern coast of China, is one of the most economically developed cities, with a resident population of 9.697 million in 2023. Ningbo city is situated in a subtropical monsoon climate zone characterized by distinct seasons. The average annual temperature is approximately 16.4 °C, the average annual sunshine duration is 1,850 hours, and the average annual precipitation is 1,480 millimeters.  The analysis included women admitted to obstetric departments in four medical centers between January 1, 2013, and December 31, 2023.  Demographic characteristics, medical records, and delivery details were obtained from hospital electronic medical records systems, with sensitive private information from all participants protected. This study was approved by the Ningbo University Medical Science Research Ethics Committee (Ethical Application Ref: SX201916). Written informed consent for participation was not required due to the retrospective nature of this study, in accordance with the national legislation and institutional requirements. |
| Participants | 6 | (*a*) *Cohort study*—Give the eligibility criteria, and the sources and methods of selection of participants. Describe methods of follow-up  *Case-control study*—Give the eligibility criteria, and the sources and methods of case ascertainment and control selection. Give the rationale for the choice of cases and controls  *Cross-sectional study*—Give the eligibility criteria, and the sources and methods of selection of participants | 4 | The analysis included women admitted to obstetric departments in four medical centers between January 1, 2013, and December 31, 2023. The inclusion criteria for all the subjects were as follows: aged 20– to 49 years, singleton pregnancies, natural conception, gestational week of at least 28 weeks, availability of diagnostic information for mothers, continuous residence during pregnancy, and Han Chinese ethnicity. Pregnant women diagnosed with diabetes, heart disease, kidney disease, severe liver disease, cancer, miscarriage, or missing last menstrual period (LMP) data before pregnancy were excluded from the study.  A total of 92,332 pregnant women were ultimately included in the analysis. Gestational weeks were determined by the last menstrual period date and ultrasound examination. Conception seasons (spring: March–May, summer: June–August, autumn: September–November and winter: December–February) were calculated based on the last menstrual period. |
| Variables | 7 | Clearly define all outcomes, exposures, predictors, potential confounders, and effect modifiers. Give diagnostic criteria, if applicable | 4-5 | In this study, four common pregnancy complications were included in the analysis as outcomes: GDM, GH, PE, and hypothyroidism.  The GDM diagnosis is accomplished with the “one-step” 75-g oral glucose tolerance test (OGTT) derived from the International Association of the Diabetes and Pregnancy Study Groups (IADPSG) GDM diagnostic criteria (McIntyre et al., 2016). A 75-g OGTT, with plasma glucose measurements when the individual is fasting and at 1 and 2 h, is performed at 24–28 weeks of gestation in individuals not previously diagnosed with diabetes. The OGTT should be performed in the morning after an overnight fast of at least 8 h. The diagnosis of GDM is made when any of the following plasma glucose values are met or exceeded: fasting glucose higher than or equal to 92 mg/dL (5.1 mmol/L), 1 h blood glucose higher than or equal to 180 mg/dL (10.0 mmol/L), and 2 h blood glucose higher than or equal to 153 mg/dL (8.5 mmol/L).  GH is new-onset hypertension at ≥20 weeks of gestation in the absence of proteinuria or other findings suggestive of preeclampsia. PE is gestational hypertension with ≥1 new onset of organ or uteroplacental dysfunction: proteinuria, other maternal end-organ dysfunction, or uteroplacental dysfunction (Magee et al., 2022).  The diagnosis of hypothyroidism during pregnancy is based on the serum levels of thyroid-stimulating hormone (TSH) and free thyroxine (FT4). Clinical hypothyroidism (CH) is defined as a TSH level higher than the upper limit of the specific reference range (or 4.0 mU/L in the first trimester) and an FT4 level less than the lower limit of the specific reference range during pregnancy. Subclinical hypothyroidism (SCH) is defined as a TSH level higher than the upper limit of the specific reference range (or 4.0 mU/L in the first trimester) and an FT4 level in the normal range during pregnancy.  Definition of the MFS:  In this study, we calculated the MFS according to these nine meteorological factors (Wang et al., 2021). Initially, individual exposure values for the nine meteorological factors across all participants were normalized using min–max standardization. The equation:  *X_new_* = (*X* − *X_min_*) / (*X_max_* − *X_min_*)  We subsequently summed the exposure values of the nine meteorological factors and weighted them according to adjusted multivariate estimates of high-frequency risk (β coefficients) to derive the weighted MFS. The equation was as follows:  MFS = (β_Tmean_ × *T_mean_* + β_RH_ × *RH* + β_surface pressure_ × *surface pressure* + β_wind speed_ × *wind speed* + β_precipitation_ × *precipitation* + β_sunshine duration_ × *sunshine duration* + β_Tmax_ × *T_max_* + β_Tmin_ × *T_min_* + β_DTR_ × *DTR*) × (9/sum of the β coefficients)  A higher MFS indicates a greater degree of exposure to environmental meteorological factors. In addition, the participants were divided into five groups according to the quintiles of the MFS. |
| Data sources/ measurement | 8* | For each variable of interest, give sources of data and details of methods of assessment (measurement). Describe comparability of assessment methods if there is more than one group | 4-5 | Demographic characteristics, medical records, and delivery details were obtained from hospital electronic medical records systems, with sensitive private information from all participants protected.  We collected the daily mean temperature (Tmean), relative humidity (RH), surface pressure, wind speed, precipitation, sunshine duration, maximum temperature (Tmax), minimum temperature (Tmin) and DTR in Ningbo from January 1, 2013, to December 31, 2023, to evaluate the relationships between meteorological factors and these four pregnancy complications. Tmean, RH, surface pressure, wind speed, precipitation, sunshine duration, Tmax, and Tmin records were obtained from the National Aeronautics and Space Administration (NASA) Goddard Earth Sciences (GES) Data and Information Services Center (DISC) Global Land Data Assimilation System (GLDAS) (Rodell et al., 2004; Beaudoing, H. and M. Rodell). The DTR is calculated as the difference between Tmax and Tmin.  We averaged these variables for the first day of each pregnancy week and the subsequent six days to assess the average individual exposure levels of meteorological factors per week of pregnancy for each participant. We calculated the average individual exposure levels of meteorological factors for the following three periods: the first trimester (1–13 weeks), the second trimester (14–27 weeks), and the first two trimesters combined (1–27 weeks). |
| Bias | 9 | Describe any efforts to address potential sources of bias | 5-6 | We adjusted all the models for the same confounding factors, including maternal age, parity (1, 2, ≥3), gravidity (1, ≥2), season of conception (spring, summer, autumn, or winter), and year of conception. |
| Study size | 10 | Explain how the study size was arrived at | 4 | A total of 92,332 pregnant women were ultimately included in the analysis. |

Continued on next page

| Quantitative variables | 11 | Explain how quantitative variables were handled in the analyses. If applicable, describe which groupings were chosen and why | 5-6 | We used descriptive statistics to summarize the demographic characteristics of the study population and the exposure to meteorological factors during the study period. Continuous variables are presented as the means and standard deviations (means ± SDs), and categorical variables are presented as frequencies and percentages. The Pearson linear correlation test was employed to examine the relationships between the various meteorological factors.  We assessed the impact of each individual meteorological factor and MFS on pregnancy complications. Because GDM, GH and PE were mainly diagnosed in the second trimester in the study population, we used a logistic regression model to estimate the associations between each meteorological factor and MFS on the risk of GDM, GH and PE during the first trimester, the second trimester and the first two trimesters. As hypothyroidism is diagnosed mainly in the first trimester, we used a logistic regression model to estimate the associations between each meteorological factor and MFS with hypothyroidism during the first trimester. |
| --- | --- | --- | --- | --- |
| Statistical methods | 12 | (*a*) Describe all statistical methods, including those used to control for confounding | 5-7 | We used descriptive statistics to summarize the demographic characteristics of the study population and the exposure to meteorological factors during the study period. Continuous variables are presented as the means and standard deviations (means ± SDs), and categorical variables are presented as frequencies and percentages. The Pearson linear correlation test was employed to examine the relationships between the various meteorological factors. We adjusted all the models for the same confounding factors, including maternal age, parity (1, 2, ≥3), gravidity (1, ≥2), season of conception (spring, summer, autumn, or winter), and year of conception. The results for all the models are presented as odds ratios (ORs) with 95% confidence intervals (CIs).  We assessed the impact of each individual meteorological factor and MFS on pregnancy complications. Because GDM, GH and PE were mainly diagnosed in the second trimester in the study population, we used a logistic regression model to estimate the associations between each meteorological factor and MFS on the risk of GDM, GH and PE during the first trimester, the second trimester and the first two trimesters. As hypothyroidism is diagnosed mainly in the first trimester, we used a logistic regression model to estimate the associations between each meteorological factor and MFS with hypothyroidism during the first trimester.  We then assessed the effects of sensitive windows of exposure to extreme meteorological factors on the risk of pregnancy complications. Distributed lag nonlinear models (DLNMs) incorporating logistic regression were applied to estimate the exposure‒lag‒response effects of exposure to extreme meteorological factors on the risk of pregnancy complications (Gasparrini, 2014). The 95th, 97th and 99th percentiles of each meteorological variable were defined as extremely high values, whereas the 5th, 3rd and 1st percentiles were defined as extremely low values, with the median of each meteorological variable serving as the reference value (Zhang et al., 2021). Considering that GDM is diagnosed between weeks 24 and 28 of pregnancy and that GH and PE are diagnosed after week 20 of pregnancy, the lag time was set from 1– to 24 gestational weeks. Hypothyroidism is diagnosed early in pregnancy, so the lag time range was set from 1– to 13 gestational weeks. Given the potential nonlinear effects of meteorological factors, we employed natural cubic spline functions to model the delayed response relationship between exposure at each gestational week and the outcome (Wang et al., 2020). The optimal degrees of freedom were determined based on the minimum Akaike information criterion (AIC) (Gasparrini, 2014).  Finally, we evaluated the effects of extreme meteorological factors during different stages of pregnancy on the risk of pregnancy complications. Meteorological factors were categorized into three groups. The 95th, 97th, and 99th percentiles of each meteorological variable were defined as extremely high values, whereas the 5th, 3rd, and 1st percentiles were defined as extremely low values. The ranges from the 5th– to 95th percentiles, 3rd– to 97th percentiles, and 1st– to 99th percentiles were designated as reference values. Logistic regression models were used to assess whether extreme meteorological factors, compared with reference values, influenced the occurrence of pregnancy complications. For the interaction analysis, we used the relative risk owing to interaction (RERI), proportion attributable (AP) and synergy index (SI) to estimate the additive interactive effects of extreme meteorological factors on the risk of pregnancy complications in different trimesters (Lou et al., 2018; Zhang et al., 2021). Each statistically significant extreme meteorological factor was explored for its two-by-two additive interaction. If there is an additive interaction between the two factors, the RERI with 95% CI and AP with 95% CI should not include 0, whereas the SI with 95% CI should not include 1. RERI and AP > 0 and SI > 1 indicate positive interactions, whereas RERI and AP < 0 and SI > 1 indicate negative interactions.  All the statistical analyses were conducted using R software (version 4.2.3). A two-sided test with P < 0.05 was considered statistically significant. Each reported odds ratio in this study was the adjusted OR (aOR). |
|  |  | (*b*) Describe any methods used to examine subgroups and interactions |  |  |
|  |  | (*c*) Explain how missing data were addressed |  |  |
|  |  | (*d*) *Cohort study*—If applicable, explain how loss to follow-up was addressed  *Case-control study*—If applicable, explain how matching of cases and controls was addressed  *Cross-sectional study*—If applicable, describe analytical methods taking account of sampling strategy |  |  |
|  |  | (*e*) Describe any sensitivity analyses |  |  |
| Results | | | | |
| Participants | 13* | (a) Report numbers of individuals at each stage of study—eg numbers potentially eligible, examined for eligibility, confirmed eligible, included in the study, completing follow-up, and analysed | 7 | A total of 92,332 participants were ultimately included in the study. |
|  |  | (b) Give reasons for non-participation at each stage |  |  |
|  |  | (c) Consider use of a flow diagram |  |  |
| Descriptive data | 14* | (a) Give characteristics of study participants (eg demographic, clinical, social) and information on exposures and potential confounders | 7 | **Basic characteristics of the participants and exposure to meteorological factors during pregnancy**  A total of 92,332 participants were ultimately included in the study; 17,814 (19.29%) were diagnosed with GDM, 3,860 (4.18%) were diagnosed with GH, 3,101 (3.36%) were diagnosed with PE, and 17,418 (18.86%) were diagnosed with hypothyroidism (Table 1). Overall, the mean (SD) maternal age of the participants was 30.02 (4.57) years, and 49,248 (53.34%) were primiparous. In general, advanced maternal age, increased gravidity and spring pregnancy increased the risk of GDM, GH and PE (Tables S1–S3). Primiparous individuals had increased risks of GH, PE and hypothyroidism (Tables S2–S4). Residents had increased risks of GDM and GH, whereas immigrants had increased risks of GH and PE (Tables S1–S4).  Table S5 presents the average exposure levels of meteorological factors during different trimesters in patients with GDM, GH, PE and hypothyroidism and in healthy pregnant women. During the first trimester, patients with GDM, GH, PE and hypothyroidism had higher exposure levels of T_mean_, RH, precipitation, T_max_ and T_min_ and lower exposure levels of surface pressure. During the second trimester, patients with GDM, GH and PE had higher exposure levels of T_mean_, RH, T_max_ and T_min_. The correlations among the meteorological factors in the different trimesters are shown in Fig. S1. The distributions of meteorological factors during different trimesters among all participants are summarized in Table S6. In addition, the distributions of weekly meteorological factors during the first 24 and 13 gestational weeks among participants are shown in Tables S7 and S8, respectively. |
|  |  | (b) Indicate number of participants with missing data for each variable of interest |  |  |
|  |  | (c) *Cohort study*—Summarise follow-up time (eg, average and total amount) |  |  |
| Outcome data | 15* | *Cohort study*—Report numbers of outcome events or summary measures over time |  |  |
|  |  | *Case-control study—*Report numbers in each exposure category, or summary measures of exposure |  |  |
|  |  | *Cross-sectional study—*Report numbers of outcome events or summary measures |  |  |
| Main results | 16 | (*a*) Give unadjusted estimates and, if applicable, confounder-adjusted estimates and their precision (eg, 95% confidence interval). Make clear which confounders were adjusted for and why they were included | 7-9 | **Associations between meteorological factors and pregnancy complications**  Table 2 shows the associations between individual meteorological factors and pregnancy complications, including GDM, GH, PE and hypothyroidism, during different trimesters after adjusting for potential confounding factors.  During the first trimester, elevated T_mean_, T_max_ and T_min_ values were associated with increased risks of GDM (T_mean_: aOR = 1.110, 95% CI: 1.050–1.174; T_max_: aOR = 1.110, 95% CI: 1.056–1.167; T_min_: aOR = 1.107, 95% CI: 1.053–1.165), GH (T_mean_: aOR = 1.182, 95% CI: 1.060–1.319; T_max_: aOR = 1.181, 95% CI: 1.070–1.303; T_min_: aOR = 1.160, 95% CI: 1.050–1.280), PE (T_mean_ = 1.135, 95% CI: 1.006–1.280; T_max_ = 1.131, 95% CI: 1.014–1.260; T_min_: aOR = 1.133, 95% CI: 1.017–1.263) and hypothyroidism (T_mean_: aOR = 1.145, 95% CI: 1.082–1.212; T_max_: aOR = 1.092, 95% CI: 1.038–1). Greater quantities of precipitation were associated with increased risks of GH (aOR = 1.028, 95% CI: 1.006–1.050) and hypothyroidism (aOR = 1.013, 95% CI: 1.002–1.024). A longer duration of sunshine was associated with increased risks of GDM (aOR = 1.093, 95% CI: 1.050–1.138) and GH (aOR = 1.234, 95% CI: 1.141–1.335). However, increasing surface pressure was associated with lower risks of GDM (aOR = 0.895, 95% CI: 0.848–0.944), GH (aOR = 0.798, 95% CI: 0.719–0.885), PE (aOR = 0.885, 95% CI: 0.787–0.995) and hypothyroidism (aOR = 0.983, 95% CI: 0.889–0.990). An increase in RH was associated with increased risks of GDM (aOR = 1.058, 95% CI: 1.007–1.112) and PE (aOR = 1.111). 95% CI: 1.003–1.231) but was associated with a decreased risk of hypothyroidism (aOR = 0.911, 95% CI: 0.867–0.957). Wind speed was associated with a decreased risk of PE (aOR = 0.840, 95% CI: 0.709–0.995) but was associated with an increased risk of hypothyroidism (aOR = 1.211, 95% CI: 1.113–1.319).  Higher T_mean_, T_max_ and T_min_ values during the second trimester consistently increased the risk of GDM (T_mean_: aOR = 1.074, 95% CI: 1.014–1.138; T_max_: aOR = 1.061, 95% CI: 1.008–1.117; T_min_: aOR = 1.070, 95% CI: 1.016–1.126) and GH (T_mean_: aOR = 1.316, 95% CI: 1.177–1.472; T_max_: aOR = 1.264, 95% CI: 1.143–1.397; T_min_: aOR = 1.265, 95% CI: 1.144–1.399). Similarly, increased RH during the second trimester was also related to the risk of GDM (aOR = 1.054, 95% CI: 1.001–1.110) and PE (aOR = 1.116, 95% CI: 1.003–1.242). An increase in precipitation was also associated with an increased risk of GDM (aOR = 1.014, 95% CI: 1.003–1.026). Increases in surface pressure and DTR were associated with decreased risks of GH (aOR = 0.832, 95% CI: 0.748–0.925) and PE (aOR = 0.925, 95% CI: 0.874–0.978), respectively.  We also constructed the MFS to assess the effects of comprehensive exposure to meteorological factors on pregnancy complications. Table 3 showed that compared with participants with MFS in the lowest quintile, participants with MFS in the highest quintile had greater risks of GDM (aOR = 1.186, 95% CI: 1.079–1.304), GH (aOR = 1.596, 95% CI: 1.323–1.925), PE (aOR = 1.347, 95% CI: 1.094–1.658) and hypothyroidism (aOR = 1.257, 95% CI: 1.141–1.385). In the second trimester, compared with participants with MFS in the lowest quintile, participants with MFS in the highest quintile had greater risks of GDM (aOR = 1.124, 95% CI: 1.026–1.231) and GH (aOR = 1.382, 95% CI: 1.151–1.659) but a lower risk of PE (aOR = 0.800, 95% CI: 0.673–0.952). In the first two trimesters, compared with participants with MFS in the lowest quintile, participants with MFS in the highest quintile had greater risks of GDM (aOR = 1.221, 95% CI: 1.078–1.383), GH (aOR = 1.413, 95% CI: 1.101–1.813) and PE (aOR = 1.248, 95% CI: 1.010–1.542). All P values for trends were < 0.05. The results were consistent with the aORs (95% CI) of pregnancy complications per 10-point increase in MFS (Table S9). These results suggest that the risk of pregnancy complications increases with higher levels of exposure to risky meteorological factors in the first and second trimesters.  **Effects of extreme meteorological factors on the risk of pregnancy complications**  Tables S12–S15 show that extreme meteorological factors were associated with the risk of all four pregnancy complications in different trimesters. Figure 1 shows susceptible windows of extremely high meteorological factors for GDM, GH and PE during the first 24 gestational weeks and the risk of hypothyroidism during the first 13 gestational weeks. Compared with the median meteorological exposure, extremely high precipitation and sunshine duration were associated with increased risks of GDM during the 13th–20th and 1st–8th gestational weeks, respectively. Extremely high T_mean_, RH, wind speed, precipitation, sunshine duration and T_max_ were associated with increased risks of GH during the 18th–23rd, 15th–18th, 18th, 5th–19th, 6th–8th and 18th–21st gestational weeks, respectively. Extremely high RH, surface pressure, precipitation, sunshine duration and T_max_ were associated with increased risks of PE during the 4th–9th, 9th–10th, 1st–7th or 18th–24th, 6th–20th and 10th–13th or 23rd–24th gestational weeks, respectively. Extremely high T_mean_, RH, surface pressure, wind speed, precipitation, sunshine duration and T_max_, T_min_ and DTR were associated with increased risks of hypothyroidism during the 2nd–6th, 1st–8th, 2nd–13th, 2nd–12th, 1st–13th, 10th–13th, 1st–8th, 2nd–4th and 2nd–4th gestational weeks, respectively. No significant effects of other extremely high meteorological factors on the risk of pregnancy complications were observed (Table S10). In conclusion, the sensitive time windows of extremely high meteorological factors on the risks of GDM, GH and PE were mainly in the 3rd– to 5th months of pregnancy, and the sensitive time window of extremely high meteorological factors on the risk of hypothyroidism was mainly throughout the first trimester.  Figure 2 shows the windows of susceptibility for the effects of extremely low meteorological factors on the risks of GDM, GH and PE during the first 24 gestational weeks and the risk of hypothyroidism during the first 13 gestational weeks. Compared with the median meteorological factors, extremely low DTR was associated with an increased risk of GDM during the 1st gestational week. Extremely low surface pressure, wind speed and T_min_ were associated with increased risks of GH during the 11th–14th, 10th–19th and 9th–16th gestational weeks, respectively. Extremely low precipitation, T_max_ and DTR were associated with increased risks of PE during the 18th–21st, 11th–14th and 10th–19th gestational weeks, respectively. Extremely low T_mean_, RH, surface pressure, wind speed, precipitation, T_max_, T_min_ and DTR values were associated with increased risks of hypothyroidism during the 2nd–5th or 12th–13th, 1st–3rd or 12th–13th, 4th–5th, 4th–7th, 1st–13th, 1st–4th or 12th–13th, 2nd–7th or 12th–13th and 1st–3rd or 11th–13th gestational weeks, respectively. No significant effects of other extremely low meteorological factors on the risk of pregnancy complications were observed (Table S11). In summary, the sensitive time window of extremely low meteorological factors on the risk of GH and PE was mainly from the 3rd– to 5th months of pregnancy, whereas the sensitive time window of extremely low meteorological factors on the risk of hypothyroidism was mainly throughout the first trimester. |
|  |  | (*b*) Report category boundaries when continuous variables were categorized |  |  |
|  |  | (*c*) If relevant, consider translating estimates of relative risk into absolute risk for a meaningful time period |  |  |

Continued on next page

| Other analyses | 17 | Report other analyses done—eg analyses of subgroups and interactions, and sensitivity analyses | 9-10 | **Effects of interactions between extreme meteorological factors on the risk of pregnancy complications**  When we investigated the potential additive interactions between extreme meteorological factors, no statistically significant interaction effects between extreme meteorological factors and the risks of GDM, GH and PE were observed (Tables S16–S18). Table 4 shows that there were interactions between extreme meteorological factors and the risk of hypothyroidism during the first trimester. When extreme meteorological factors were defined by the 5th and 95th percentiles of meteorological factors, there were positive interactions between extremely low T_min_ (3.97 °C) and extremely low precipitation (2.36 mm) (RERI and 95% CI: 0.60 (0.23, 1.03); AP and 95% CI: 0.33 (0.13, 0.45); SI and 95% CI: 3.89 (1.68, 9.00)), extremely low T_min_ (3.97 °C) and extremely high wind speed (4.03 m/s) (RERI and 95% CI: 1.06 (0.60, 1.61); AP and 95% CI: 0.44 (0.27, 0.54); SI and 95% CI: 3.90 (2.00, 7.61)), extremely low T_min_ (3.97 °C) and extremely high DTR (8.93 °C) (RERI and 95% CI: 0.74 (0.33, 1.22); AP and 95% CI: 0.39 (0.19, 0.50); SI and 95% CI: 5.41 (1.94, 15.07)) and extremely high wind speed (4.03 m/s) and extremely high DTR (8.93 ℃) (RERI and 95% CI: 0.66 (0.29, 1.05); AP and 95% CI: 0.36 (0.17, 0.49); SI and 95% CI: 4.67 (1.14, 19.09)). When meteorological factor extremes were defined by the 3rd and 97th percentiles of meteorological factors, there were positive interactions between extremely low T_mean_ (7.74 °C) and extremely high wind speed (4.17 m/s) (RERI and 95% CI: 3.02 (1.45, 5.36); AP and 95% CI: 0.65 (0.42, 0.74); SI and 95% CI: 5.89 (2.88, 12.03)) and extremely low T_mean_ (7.74 °C) and extremely high DTR (9.07 °C) (RERI and 95% CI: 2.53 (0.69, 6.17); AP and 95% CI: 0.67 (0.23, 0.72); SI and 95% CI: 12.45 (3.61, 42.91)) and a negative interaction between extremely low precipitation (2.13 mm) and extremely high wind speed (4.17 m/s) (RERI and 95% CI: −0.73 (−1.45, −0.10); AP and 95% CI: −0.48 (−1.10, −0.08); SI and 95% CI: 0.42 (0.19, 0.92)). When meteorological factor extremes were defined by the 1st and 99th percentiles of meteorological factors, there was a positive interaction between extremely low T_mean_ (7.44 °C) and extremely high surface pressure (1023.13 hPa) (RERI and 95% CI: 2.02 (0.27, 5.07); AP and 95% CI: 0.54 (0.03, 0.68); SI and 95% CI: 3.93 (1.36, 11.37)) and negative interactions between extremely low RH (65.32%) and extremely low T_min_ (2.76 °C) (RERI and 95% CI: −1.66 (−2.69, −0.82); AP and 95% CI: −1.10 (−2.05, −0.50); SI and 95% CI: 0.23 (0.10, 0.53)) and extremely low RH (65.32%) and extremely high surface pressure (1023.13 hPa) (RERI and 95% CI: −1.09 (−2.2, −0.18); AP and 95% CI: −0.63 (−1.48, −0.12); SI and 95% CI: 0.40 (0.18, 0.89)). No other statistically significant interaction effects between meteorological factors and the risk of hypothyroidism were observed (Table S19). |
| --- | --- | --- | --- | --- |
| Discussion | | | | |
| Key results | 18 | Summarise key results with reference to study objectives | 10 | Our study systematically explored the impacts of external meteorological conditions on the reproductive health of pregnant women. In this study, we not only evaluated the independent risk effects and additive interactions of various meteorological factors during the first and second trimesters in terms of pregnancy complications but also constructed an MFS based on multidimensional meteorological factors to comprehensively assess the associations between the overall level of exposure to meteorological factors during pregnancy and pregnancy complications. We also assessed the sensitive time windows for the effects of extreme meteorological factors on the risks of different pregnancy complications.  **Conclusions**  Our study suggested that meteorological factors are critical risk factors for pregnancy complications. MFS was a better indicator for evaluating the associations between meteorological factors and pregnancy complications. Both extremely high and extremely low meteorological factors increased the risk of pregnancy complications. The sensitive time windows of extremely high and low meteorological factors for the risk of GDM, GH and PE were mainly from the 3rd to 5th months of pregnancy, whereas the sensitive time window of extremely high and low meteorological factors for the risk of hypothyroidism was mainly throughout the first trimester. Interactions between extremely meteorological factors and hypothyroidism were found. Targeted protective measures should be taken to reduce the risk of pregnancy complications. |
| Limitations | 19 | Discuss limitations of the study, taking into account sources of potential bias or imprecision. Discuss both direction and magnitude of any potential bias | 13-14 | There are several limitations in our study. We were not able to obtain the specific diagnosis date of each pregnancy complication. We had no additional information on the pregnant women, such as pre-pregnancy BMI, household income, education, maternal alcohol consumption and maternal tobacco smoking, which left us with few covariates to adjust in the models. In addition, we had no specific home address information for pregnant women, which prevented us from accurately assessing the individual exposures of pregnant women. However, we believe that meteorological factors fluctuated little in our study area, which did not affect our results. In our study, the MFS, which is based on multidimensional meteorological factors, was used for the first time to assess the correlation between environmental meteorological factors and pregnancy complications comprehensively. Based on weekly exposure levels during pregnancy, we assessed the sensitive time window for the impact of extreme meteorological factors on complications during pregnancy. Our research comprehensively reflected the impact of climate change on pregnant women's health. |
| Interpretation | 20 | Give a cautious overall interpretation of results considering objectives, limitations, multiplicity of analyses, results from similar studies, and other relevant evidence | 10-13 | The effects of meteorological factors on pregnant women have been previously reported in the literature. Booth and colleagues reported that each 10 °C increase in the mean 30-day temperature before the 27th week of pregnancy increased the GDM risk by 6% (Booth et al., 2017). Su and colleagues reported that GDM risk increased by 3% for each 1 °C increase in the mean 35-day period prior to GDM diagnosis from 14 °C to 27 °C, whereas GDM risk increased by 54% at temperatures above 28 °C. A higher prevalence of GDM was associated with a smaller difference in temperature within a day (Su et al., 2020). Some studies have reported that extreme meteorological factors are also linked to GDM risk. Teyton and colleagues reported that GDM risk increased with extremely low temperatures from the 20th– to 24th gestational weeks and with extremely high temperatures from the 11th– to 14th gestational weeks (Teyton et al., 2023). Zhang and colleagues reported that the time window of susceptibility to the effects of extreme temperatures and DTR on GDM occurred in the second trimester (Zhang et al., 2021). Our findings are partially consistent with these studies, which revealed that not only mean temperature but also other meteorological factors, including RH, surface pressure, precipitation, sunshine duration, T_max_ and T_min_, were associated with the risk of GDM during the first and second trimesters in the present study. However, there was no association between extreme temperature and GDM risk in this study. In our study, GDM risk increased with extremely high precipitation from the 13th– to 20th gestational weeks, sunshine duration from the 1st– to 8th gestational weeks, and extremely low DTR during the 1st gestational week.  There are several biological hypotheses to explain the influence of meteorological factors on the risk of GDM. Brown adipose tissue (BAT) plays an important role in glucose metabolism. Cold exposure can increase BAT activation, which improves whole-body glucose homeostasis and insulin sensitivity (van der Lans et al., 2013; Chondronikola et al., 2014). High-temperature exposure reduces insulin sensitivity. High temperatures may also lead to beta-cell dysfunction in pregnant women, which reduces insulin sensitivity (Retnakaran et al., 2018). Moses et al. reported the acute effect of ambient temperature on apparent glucose tolerance and concluded that this effect was due to changes in core body temperature leading to the redistribution of blood flow between the cutaneous and visceral beds (Moses et al., 1997). Adequate vitamin D levels may reduce the risk of GDM (Rizzo et al., 2019). Skin exposure to solar ultraviolet B radiation is a major source of vitamin D (Saraff & Shaw, 2016). High ambient temperatures, high precipitation and low sunshine duration will make people go out less, reducing the opportunity to receive sunlight, leading to insufficient synthesis of vitamin D in the body. Furthermore, relative dehydration of the body caused by high temperature leads to hemoconcentration, causing an elevation in blood glucose levels (Preston et al., 2020). The DTR is an indicator that can reflect weather stability. A review revealed that DTR is significantly associated with human mortality and morbidity, especially cardiovascular and respiratory diseases (Cheng et al., 2014). However, extremely low DTR was associated with an increased risk of GDM in our study. We believe that greater temperature fluctuations may increase insulin sensitivity; therefore, it is necessary to study the potential effects of temperature fluctuations on metabolic system diseases.  Previous studies have also shown associations between meteorological factors and the risks of GH and PE. Xiong and colleagues reported that an increased T_mean_ during the first half of pregnancy increased the risk of PE or eclampsia and GH. Under extreme temperatures, extremely cold exposure during the first half of pregnancy decreased the risk of PE, eclampsia or GH, whereas extremely heat exposure increased the risk (Xiong et al., 2020). Zeng and colleagues reported that extremely high temperatures (aOR = 1.24, 95% CI: 1.12–1.38) and moderately high temperatures (aOR = 1.22, 95% CI 1.10–1.35) during the first trimester were associated with increased risks of PE (Zeng et al., 2024). Zhao and colleagues reported that low-temperature exposure was a significant risk factor for preeclampsia risk (Zhao, Long & Lu, 2022). Overall, the results of the effects of temperature exposure on GH and PE risk were inconsistent. In our study, every 10 °C increase in T_mean_, T_max_ or T_min_ during the first two trimesters was associated with an increased risk of GH. A 10 °C increase in T_mean_, T_max_ or T_min_ during the first trimester was associated with an increased risk of PE. Under extreme temperatures, both extremely high and extremely low temperatures were associated with the risks of GH and PE. Extremely high temperatures increased the risks of GH and PE during the second trimester, whereas extremely low temperatures increased the risks of GH and PE mainly during the first trimester.  The mechanisms underlying the effects of temperature exposure on HDP risk are unclear. Some studies have suggested that the pathogenesis of preeclampsia or eclampsia and gestational hypertension are similar (Xiong et al., 2020). As pregnancy progresses, the increasing weight of the pregnant woman and the need for fetal growth lead to a decrease in maternal heat loss and an increase in internal heat production. Thus, heat exposure could disrupt thermoregulation and increase the risks of GH and PE (Mao et al., 2023). When exposed to cold temperatures, both the sympathetic nervous system and the renin–angiotensin system are activated, which could cause vasoconstriction and an increase in blood pressure (Park et al., 2020). In addition, cold exposure induces endothelial dysfunction. Thus, cold exposure is also a risk factor for HDP. It seems that the impact of extreme temperatures on HDP risk is bidirectional. Vitamin D benefits maternal health. Adequate vitamin D is important for reducing the risk of GDM, GH, PE and other complications (Zhang et al., 2022). In our study, extremely high RH, precipitation and sunshine increased the risks of both GH and PE. We believe that extremely high humidity, precipitation and sunshine duration lead to reduced outdoor activity, resulting in insufficient vitamin D production by the body.  The thyroid gland is an important endocrine organ that regulates metabolism by secreting thyroid hormones (THs). TH production and release are regulated by the hypothalamic‒pituitary‒thyroid axis (Chaker et al., 2022). Thus, the thyroid gland is very sensitive to changes in ambient temperature. Few studies have investigated meteorological factors and the risk of hypothyroidism. In our study, the impact of extreme temperatures in the first trimester on the risk of hypothyroidism was bidirectional. Both extremely high and extremely low temperatures increased the risk of hypothyroidism. We believe that extreme temperatures affect the regulation of the hypothalamic‒pituitary‒thyroid axis. Almost all extreme meteorological factors are associated with an increased risk of hypothyroidism. Hypothyroidism may be more susceptible to meteorological factors in early pregnancy than other pregnancy complications. Vitamin D deficiency may be associated with an increased risk of thyroid autoimmunity (Nettore et al., 2017). Extreme weather can reduce people's ability to perform outdoor activities, which leads to insufficient synthesis of the sunshine vitamin.  Our study revealed that pregnant women were more vulnerable to meteorological factors in the first trimester than in the second trimester, which increased the risk of pregnancy complications. This is consistent with the conclusions reached by others (Zeng et al., 2024). Early pregnancy is a critical period for embryo implantation, vascularization and placentation (Xiong et al., 2020). Dramatic changes in hormone levels in pregnant women during early pregnancy and changes in the external environment, including changes in meteorological factors, may interfere with these processes and lead to complications during pregnancy (Dreiling, Carman & Brown, 1991; Wang et al., 2024).  With respect to extreme temperatures, both extremely high and extremely low meteorological factors increased the risk of pregnancy complications, but extremely high meteorological factors had greater impacts. GDM, GH, PE and hypothyroidism are all endocrine diseases that occur during pregnancy. Environmental heat exposure affects many hormones associated with adaptation to heat, including cortisol, thyroid hormones, arginine vasopressin (AVP), prolactin, growth hormone (GH), insulin, and adipokines. High temperature affects thermoregulation and contributes to the development of endocrine diseases by inducing a stress hormone response, inhibiting the activity of the thyroid axis, regulating the body's water balance, affecting sweating and heat loss, affecting insulin sensitivity, and affecting adipose tissue (Hannan et al., 2024). Thus, appropriate meteorological conditions may reduce the risk of pregnancy complications for pregnant women. |
| Generalisability | 21 | Discuss the generalisability (external validity) of the study results | 13 | To our knowledge, this is the first study to use the MFS metric to assess its association with the risk of pregnancy complications. In our study, MFS was significantly associated with the risk of all four pregnancy complications. Our constructed MFS metrics were better predictors of the risk of pregnancy complications.  This study is the first to explore the effects of interactions between meteorological factors and the risk of pregnancy complications. We detected interactions between extreme meteorological factors and the risk of hypothyroidism, and the interactions were mainly between extreme temperature-related variables, including extremely low Tmean, extremely low Tmin and extremely high DTR, and other meteorological factors.  The impact of meteorology on the reproductive health of pregnant women is complex; therefore, we need to take a multifaceted and holistic view of its role. More epidemiologic and physiologic studies are needed to investigate the associations between temperature and other meteorological factors and pregnancy complications and their mechanisms of effects. |
| Other information | |  | | |
| Funding | 22 | Give the source of funding and the role of the funders for the present study and, if applicable, for the original study on which the present article is based | / | This work was supported by Beijing Zhongwei Joint Funds of the Zhejiang Provincial Natural Science Foundation of China (No. LBY24H040001 and No. LBY24H040002), Zhejiang Medicine and Health Science and Technology Project (No. 2024XY149), Ningbo Youth Science and Technology Innovation Leaders Project (No. 2023QL057) and Ningbo Key Research and Development Project (No. 2024Z223 and No. 2024Z225). |

*Give information separately for cases and controls in case-control studies and, if applicable, for exposed and unexposed groups in cohort and cross-sectional studies.

**Note:** An Explanation and Elaboration article discusses each checklist item and gives methodological background and published examples of transparent reporting. The STROBE checklist is best used in conjunction with this article (freely available on the Web sites of PLoS Medicine at http://www.plosmedicine.org/, Annals of Internal Medicine at http://www.annals.org/, and Epidemiology at http://www.epidem.com/). Information on the STROBE Initiative is available at www.strobe-statement.org.
